# Spatial control of self-organizing vascular networks with programmable aptamer-tethered growth factor photopatterning

**DOI:** 10.1016/j.mtbio.2023.100551

**Published:** 2023-01-20

**Authors:** Deepti Rana, Prasanna Padmanaban, Malin Becker, Fabian Stein, Jeroen Leijten, Bart Koopman, Jeroen Rouwkema

**Affiliations:** a Department of Biomechanical Engineering, Technical Medical Centre, Faculty of Engineering Technology, University of Twente, 7522NB Enschede, the Netherlands; b Department of Developmental BioEngineering, Faculty of Science and Technology, Technical Medical Centre, University of Twente, 7522NB Enschede, the Netherlands

**Keywords:** Aptamers, Vascular endothelial growth factor, Photopatterning, Growth factor delivery, Self-organizing vasculature, Tissue engineering

## Abstract

Given the dynamic nature of engineered vascular networks within biofabricated tissue analogues, it is instrumental to have control over the constantly evolving biochemical cues within synthetic matrices throughout tissue remodeling. Incorporation of pro-angiogenic vascular endothelial growth factor (VEGF_165_) specific aptamers into cell-instructive polymer networks is shown to be pivotal for spatiotemporally controlling the local bioactivity of VEGF that selectively elicit specific cell responses. To harness this effect and quantitatively unravel its spatial resolution, herein, bicomponent micropatterns consisting of VEGF_165_ specific aptamer-functionalized gelatin methacryloyl (GelMA) (aptamer regions) overlaid with pristine GelMA regions using visible-light photoinitiators (Ru/SPS) were fabricated via two-step photopatterning approach. For the 3D co-culture study, human umbilical vein-derived endothelial cells and mesenchymal stromal cells were used as model cell types. Bicomponent micropatterns with spatially defined spacings (300/500/800 ​μm) displayed high aptamer retention, aptamer-fluorescent complementary sequence (CS_F_) molecular recognition and VEGF sequestration localized within patterned aptamer regions. Stiffness gradient at the interface of aptamer and GelMA regions was observed with high modulus in aptamer region followed by low stiffness GelMA regions. Leveraging aptamer-tethered VEGF's dynamic affinity interactions with CS that upon hybridization facilitates triggered VEGF release, co-culture studies revealed unique characteristics of aptamer-tethered VEGF to form spatially defined luminal vascular networks covered with filopodia-like structures *in vitro* (spatial control) and highlights their ability to control network properties including orientation over time using CS as an external trigger (temporal control). Moreover, the comparison of single and double exposed regions within micropatterns revealed differences in cell behavior among both regions. Specifically, the localized aptamer-tethered VEGF within single exposed aptamer regions exhibited higher cellular alignment within the micropatterns till d5 of culture. Taken together, this study highlights the potential of photopatterned aptamer-tethered VEGF to spatiotemporally regulate vascular morphogenesis as a tool for controlling vascular remodeling *in situ.*

## Graphical abstract

**Figure:**
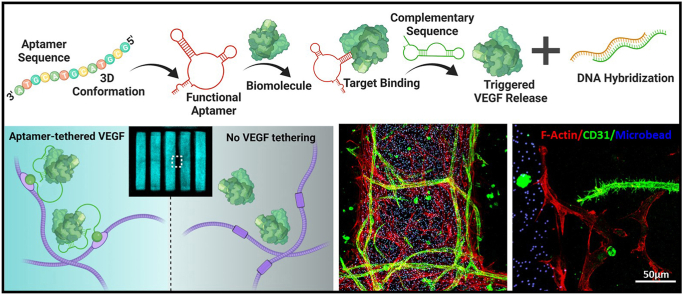

## 1 Introduction

Bioengineering tissue analogues have emerged as an alternative approach to address the scantly accessible heterologous donor tissue/organs for organ transplantation. However, lack of effective strategies to vascularize such tissue analogues has proven to be limiting their successful implementation. Engineering anastomosable, hierarchically organized, perfusable, and mature vasculature within these tissue analogues is pivotal for their survival post-implantation [1]. In nature, angiogenic processes synergistically orchestrate vascular organization and remodeling during embryonic development and afterwards. Angiogenesis can be defined as formation of new vessels from an existing vascular network either by sprouting (endothelial cells sprouts from pre-existing vessels) or intussusceptive angiogenesis (insertion of tissue pillars within existing vessels leading to its splitting) [2]. Being a multiphase process, angiogenesis is regulated spatiotemporally by various pro-angiogenic biochemical cues [2]. Vascular endothelial growth factor (VEGF), being one of the key players in angiogenesis, has been shown to stimulate distinct responses in endothelial cells such as proliferation, migration, differentiation and survival. Notably, angiogenesis employs multiple angiogenic growth factors throughout its different stages, for example, VEGF is used to promote endothelial cells migration and proliferation to initiate new vessels formation, followed by other growth factors such as platelet-derived growth factor (PDGF-BB) and angiopoietins for vessel stabilization and maturation [3]. The dynamic nature of angiogenesis displaying complex and interdependent cell-extracellular matrix (ECM) interaction highlights the need to develop sophisticated growth factor delivery systems that could spatiotemporally control multiple growth factor's presentation within synthetic ECM in a dynamic fashion.

Novel biofabrication techniques such as photolithography and 3D bioprinting have been previously used to pattern the combination of cells and/or bioactive molecules in an attempt to emulate the complex biochemical cues present in angiogenesis and fabricate macroscale tissue analogues [4]. Although previous studies have shown promising results in successfully engineering perfusable microvasculature within engineered tissues, controlling the vascular remodeling post-implantation remains a major unresolved challenge. It has been previously shown that the anastomosed vascular networks with diameters ranging from 25 to 250 ​μm that were functional initially, showed reduced vessel diameter of 10–15 ​μm upon implantation due to vascular remodeling *in vivo* [5]. These results point out that even though novel biofabrication techniques can provide control over initial vessel organization, they often overlook to account for remodeling processes that alter the initial organization either during *in vitro* culture or post-implantation. Therefore, to provide long-term functionality to the designed vasculature, it is important to have spatial as well as temporal control over local cues to guide the vascular remodeling processes.

Having spatiotemporal control over local biochemical cues (i.e., growth factors) within 3D microenvironment provides unique opportunities for eliciting cell selective responses such as adhesion, proliferation, migration, and differentiation, throughout the process of tissue maturation and remodeling *in vivo* [3]. Several attempts have been made to exploit various external stimuli including ultrasound, light, temperature, magnetic fields, electric potentials, pH, enzymes, abiotic avidin affinity, or traction forces for controlling growth factor delivery in real time [[6], [7], [8], [9], [10]]. All of these approaches have their own merits and have been widely used to accomplish release of single or multiple growth factors. These approaches, however, have struggled with specific multiple growth factor loading and the control of individual release profiles. To design advanced growth factor delivering systems that can mimic complex processes such as angiogenesis, these properties are crucial.

**Fig. 1:**
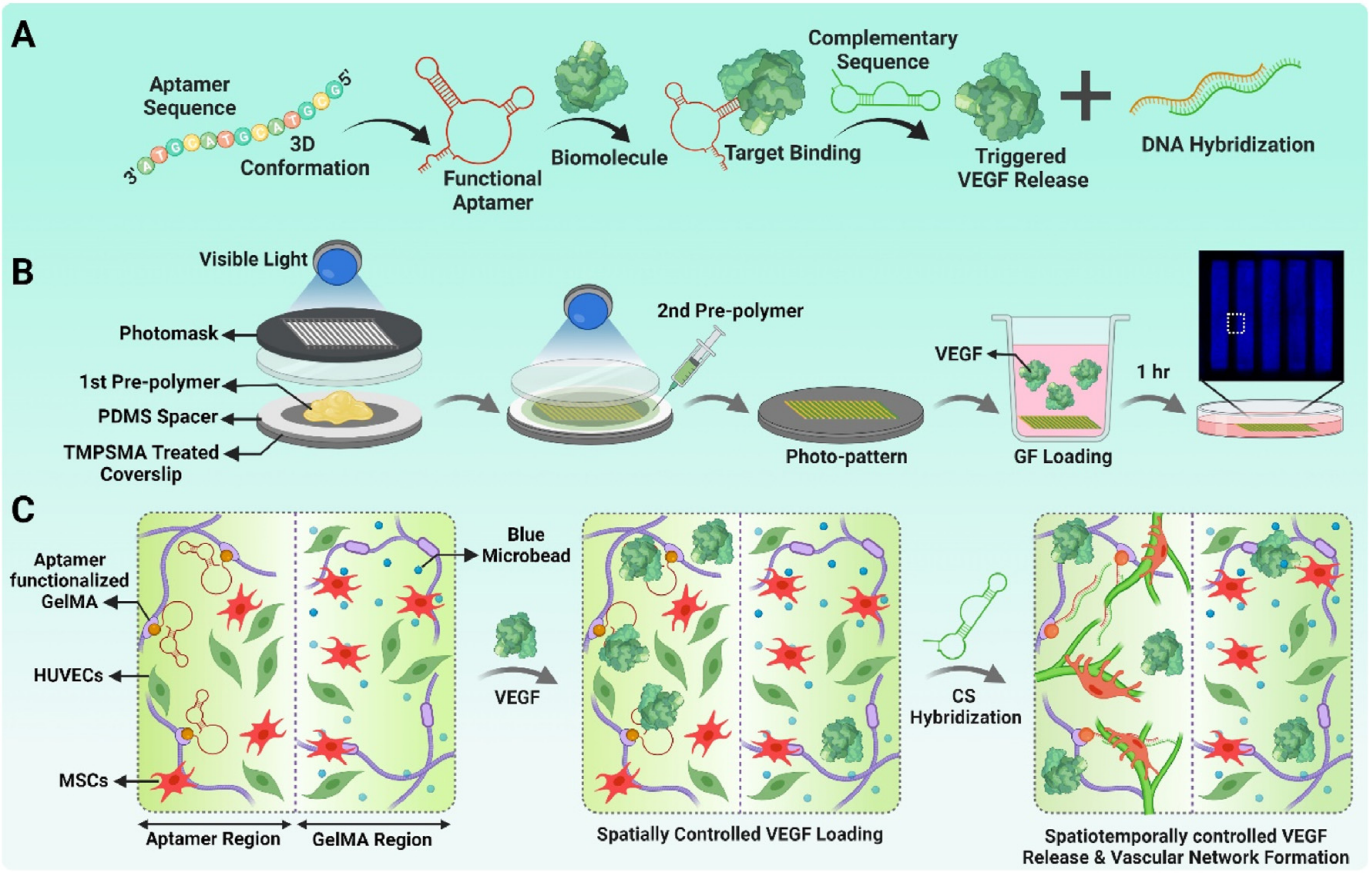
**Bicomponent micropatterns with spatially defined programmable aptamer-tethered VEGF presentation.** (A) Concept of aptamer-mediated growth factor release where aptamers specifically bind to target growth factor with high affinity, but can be programmed for growth factor release via aptamer-CS intermolecular hybridization. (B) Schematic representation of bicomponent micropattern's fabrication with two-step photocrosslinking method, followed by localized VEGF sequestration from the culture medium within 1hr due to aptamer's high affinity towards VEGF. (C) Schematic illustration highlighting two distinct regions within HUVECs/MSCs co-cultured bicomponent micropatterns where high amount of aptamer-tethered VEGF remains localized within “aptamer region”, in contrast to “GelMA region” where low VEGF concentration diffusion occurs. This disparity of VEGF concentration among both regions of micropattern and the event of triggered VEGF release upon CS hybridization, altogether selectively guides the vascular network formation, maturation and remodeling.

Leveraging on aptamer's unique capabilities, aptamer-based growth factor delivery systems have previously been shown to enable highly selective sequestration and on-demand release for multiple growth factors with high spatiotemporal control [11,12]. Aptamers are small, single-stranded oligonucleotides that are selected from synthetic RNA/DNA libraries by using SELEX technique. Based on their sequence, aptamers form 3D conformations that can selectively bind to a target with high specificity and affinity. Interestingly, the aptamer-growth factor hybridization can be reversed in the presence of complementary sequence (CS) that have higher affinity towards aptamer than the growth factor (Fig. 1A). This way, the aptamer-CS binding via intermolecular hybridization enables on-demand, programmable growth factor release using CS as an external trigger [13]. Previous studies have confirmed the ability of bulk as well as patterned aptamer-functionalized hydrogels (or polymeric particles) to sequentially release multiple growth factors via aptamer-CS hybridization [14,15]. The spatially heterogeneous aptamer distribution or gradients with different oligonucleotides have been shown to accentuate their spatiotemporal control over multiple growth factor's sequestration and release, highlighting their unique property of dynamic growth factor presentation within engineered matrices [[16], [17], [18]]. However, the direct bioactivity of aptamer-tethered growth factors within 3D matrices and its effect on guiding cellular organization such as vascular morphogenesis has scarcely been investigated or used.

Controlling cellular fate and functionality by modulating the local availability of matrix-bound signaling molecules such as growth factors within engineered tissues provides unique opportunities for *in situ* manipulation of cell behavior. For aptamer-functionalized hydrogels, this is governed by aptamer-tethered as well as soluble, free growth factor molecules. To quantitatively unravel the role of these parameters, we herein explored photopatterned cell-instructive programmable growth factor delivery platforms based on VEGF_165_-specific acrydite-modified aptamers crosslinked within biofabrication friendly, gelatin methacryloyl (GelMA) matrices. The approach refers to a recent own study where bulk patterning of aptamer-tethered VEGF molecules within cell-laden bi-phasic hydrogels unraveled the importance of spatial distribution of aptamer-tethered VEGF molecules in eliciting cell selective responses as well as guiding endothelial tubulogenesis within hydrogel matrices [13]. To explore the complete potential of this programmable, aptamer-based approach, it is important to systematically study the effect of heterogenous spatial distribution of aptamer-tethered VEGF molecules on the encapsulated cells. Harnessing the full potential of aptamer-tethered growth factor delivery platforms would not only prove as a tool for *in-situ* manipulation of cell behavior but could also guide the tissue remodeling process.

## 2 Materials and methods

### 2.1 Materials

The materials used are described in **section S1** (Supporting Information).

### 2.2 GelMA synthesis

GelMA was synthesized as described previously with medium degree of methacryloyl substitution (∼60%) [13,19]. Briefly, MA (1.25% *v/v*) was added into gelatin solution (10% *w/v*) at 0.5 ​ml/min speed for 1hr (50 ​°C). Afterwards, the reaction was diluted 5-fold with DPBS (40 ​°C) and the solution was dialyzed for 1week with 12–14 ​kDa dialysis tubing (40 ​°C) to remove residual salts. The solution was freeze-dried for 1week and stored at −20 ​°C.

### 2.3 Bicomponent micropatterns

Two-step photopatterning method was employed to pattern VEGF_165_ specific acrydite-modified aptamer-functionalized (Table S1, Supporting Information), and GelMA hydrogels (hereafter referred as *aptamer & GelMA regions*) within bicomponent micropattern. TMSPMA-treated glass slides were prepared as per the protocol described elsewhere [19], to introduce terminal acrylate functional groups on the glass surface, followed by sterilization using autoclave. To pattern aptamer region, GelMA solution (50 ​μl, 5% *w/v)* containing aptamers (25 ​μM) and visible light photoinitiator, tris(2,2′-bipyridyl)dichloro-ruthenium(II) hexahydrate and sodium persulfate (Ru/SPS, 1/10 ​mM), was added between spacers (2 ​mm) on a petri dish, covered with TMSPMA-treated glass slide (Fig. 1B), and photomask film (Fig. S1, Supporting Information) to photocrosslink with visible light (400–450 ​nm, 50 ​mW/cm^2^) for 14s. Previously reported optimized concentration of Ru/SPS for GelMA hydrogels were used [20]. Subsequently, the patterns were retrieved using warm DPBS and GelMA region was overlaid on the patterned aptamer region using GelMA pre-polymer [GelMA (5% *w/v)* ​+ ​fluorescent blue microbeads (2drops/ml) ​+ ​Ru/SPS(1/10 ​mM)] added into spacers (2 ​mm) to photocrosslink with visible light (50 ​mW/cm^2^) for 30s. The bicomponent micropatterns showed GelMA regions filled in-between the patterned aptamer region that could spatially localize aptamer-tethered VEGF molecules (Fig. 1B and C). For photopatterning UT logo, the photomasks with UT design (line width ​= ​500 ​μm) were used. Similarly, micropattern designs with constant line width (500 ​μm) but varying spacings (300/500/800 ​μm) were prepared (Fig. S1).

To investigate the effect of double exposure (or crosslinking) among the patterned regions, micropatterns with two variations were prepared. In first variation, aptamer pre-polymer (& with cells) was first photocrosslinked for 14s, followed by GelMA pre-polymer (& with cells) photocrosslinking for 30s, resulting into exposure of aptamer regions to double photocrosslinking (44s) compared to single crosslinked GelMA regions (30s). These samples were hereafter referred as “A_2_G_1_” where A_2_ stands for double crosslinked aptamer region and G_1_ for single crosslinked GelMA region. Similarly, “G_2_A_1_” samples were prepared with double crosslinked GelMA region and single-crosslinked aptamer region. As control, GelMA pre-polymer with fluorescent microbeads (& with cells) were first photopatterned, followed by overlaying plain GelMA pre-polymer (& with cells) to fill space in-between the pattern, hereafter referred as “G_B2_G_1_” samples where G_B2_ stands for double crosslinked blue fluorescent microbeads mixed GelMA region and G_1_ for single crosslinked GelMA region. Subsequently, all micropatterns were incubated with VEGF (10 ​ng) for 1hr, washed and cultured till d10.

### 2.4 Evaluation of aptamer's functionality and its retention

To evaluate aptamer incorporation and their retention, A_2_G_1_ micropatterns (aptamer line-500 μm & GelMA line-500 μm) were prepared, and incubated with Alexa Fluor-488 labelled complementary sequence (CS_F_) [13], (25 ​μM) in 1 ​ml DPBS for 24 ​h ​s at 37 ​°C. The supernatant was discarded followed by washing with DPBS to remove unbound CS_F_, supplemented with DPBS (1 ​ml) and imaged using fluorescence microscope (EVOS M7000, ThermoFisher Scientific). The experiment was carried out till d10 where after every 24hr, the sample's supernatant was replenished with fresh DPBS (1 ​ml), imaged (60% intensity, 120 ​ms exposure time: 2× objective) and sequestered CS_F_'s fluorescence intensity quantification was performed using ImageJ software (NIH, USA). Additionally, samples were imaged using confocal microscopy (Nikon A1, NIS-Elements C) on d10.

### 2.5 Evaluation of micromechanical behavior using nanoindentation

The micropatterns were investigated for their micromechanical behavior using a nano-indenter (Pavone, Optics11 Life). The G_2_A_1_ (aptamer line-500 μm & GelMA line-500 μm) & G_B2_G_1_ (GelMA with blue beads line-500 μm & GelMA line-500 μm) micropatterns were prepared and transferred to 6-well plates supplemented with DPBS (1 ​ml). As controls, bulk hydrogels using pre-polymer solutions (50 ​μl) of GelMA and GelMA mixed with fluorescent microbeads (2 drops/ml) were photocrosslinked onto the TMPSMA-treated slides using visible light (50 ​mW/cm^2^) for 44s, respectively. The samples were incubated at 37 ​°C for 24 ​h ​s to reach swelling equilibrium and then analyzed using nanoindentation with a probe of 22.5 ​μm radius tip and 0.27 ​N/m cantilever stiffness, indenting at a speed of 1 ​μm/s. The Young's modulus was determined by fitting the resulting load curves with a Hertzian contact model (assuming a Poisson ratio of 0.5) and the data fitted with r^2^ ​≤ ​0.95 was excluded. For surface mapping the Young's modulus across the micropattern, matrix scans were performed with a step width of 100 μm/step. All measurements were performed on three separate lines with at least 10 indentations per line for both regions submerged in DPBS (1 ​ml) at 37 ​°C.

### 2.6 Growth factor loading and its visualization

For growth factor sequestration, the A_2_G_1_ micropatterns (aptamer line-500 μm & GelMA line-500 μm) were prepared, transferred to ultra-low attachment 6-well plates and incubated with 1 ​ml of releasing medium (0.1% BSA in DPBS) supplemented with VEGF (10 ​ng) for 1hr at 37 ​°C (aptamer to VEGF mole ratio of ∼10,000:1). Afterwards, the supernatant was discarded, washed once and incubated with releasing medium (1 ​ml) for 24hr at 37 ​°C. Furthermore, the micropatterns were immunostained with recombinant Alexa Fluor 647 Anti-VEGFA antibody (1:100 dilution), incubated for 24hr at 4 ​°C and imaged using confocal microscopy. The maximum projection image of confocal Z-stacks were used to quantify the mean fluorescence intensity of VEGF in both regions of the micropattern using ImageJ software.

### 2.7 In-silico simulation model

A reaction diffusion-model was used to predict the free VEGF transport among both regions (aptamer & GelMA) of the micropattern. Particularly, the model was applied to analyze the free VEGF molecules distribution from the VEGF loaded aptamer region to its neighboring GelMA regions upon CS triggering (or CS absence), and to determine the changes in spatiotemporal profile of free VEGF within both regions. All the COMSOL model details, parameters, assumptions, and equations relevant to generate the simulation data are described in **section S2** and Table S2 (Supporting Information).

### 2.8 Cell culture

Human umbilical vein-derived endothelial cells (HUVECs) were cultured in EGM-2 medium with 1% (*v/v*) Pen/Strep. For human mesenchymal stromal cells (MSCs), α-MEM medium supplemented with 10% (*v/v*) FBS, 2 ​× ​10^−3^ ​M l-glutamine, 0.2 ​× ​10^−3^ ​M ascorbic acid, 1% (*v/v*) Pen/Strep and 1 ​ng/ml bFGF was used. Both cell types were cultured in a humidified atmosphere with 5% CO_2_ at 37 ​°C, medium changed every 2 ​d and passaged till 80% confluence.

### 2.9 3D Co-culture within micropatterns

For HUVECs/MSCs co-culture experiments, MSCs medium (α-MEM, 10% FBS, 2 ​mM l-glutamine, 0.2 ​mM abscorbic acid & 1% Pen/Strep) and HUVECs medium (EGM-2 Basal medium ​+ ​1% Pen/Strep ​+ ​all EGM-2 supplements except for VEGF_165_ (C-30260, PromoCell)) without the VEGF supplement in 1:1 ratio was used as co-culture medium. In all experiments, both cell types between passage 3 and 6 were used. For 3D co-culture, HUVECs (P3) and MSCs (P4) were trypsinized, counted (using trypan blue) and re-suspended in 1:1 ratio to a total seeding density of 2.5 ​× ​10^6^ ​cells/ml. The cell suspension was then centrifuged at 300 ​g for 3 ​min ​s at 4 ​°C to form the cell pellet. The supernatant was carefully removed following the addition of aptamer pre-polymer solution (5% GelMA ​+ ​25 ​μM aptamer solution ​+ ​Ru/SPS 1/10 ​mM in DPBS) and GelMA pre-polymer solution (5% GelMA ​+ ​blue fluorescent microbeads ​+ ​Ru/SPS 1/10 ​mM), respectively. The prepared aptamer and GelMA pre-polymer solutions mixed with cells were used to fabricate the A_2_G_1_, G_2_A_1_ & G_B2_G_1_ micropatterns as described previously in Section 2.3. The cell-laden micropatterns were incubated with co-culture medium (1 ​ml) supplemented with VEGF (10 ​ng) at 37 ​°C for 1hr, followed by supernatant removal, samples washed twice with DPBS and then replenished with co-culture medium (1 ​ml, without VEGF). The medium was changed after every 24hr throughout the study. To study the effect of triggered VEGF release on the encapsulated cells, 25 ​μM of CS (aptamer to CS ratio of 1:1) was added into the co-culture medium (1 ​ml) on d4.

### 2.10 Cell viability assay

Cell viability within A_2_G_1_ micropattern (aptamer line-500 μm & GelMA line-500 μm) was evaluated using Live/Dead assay kit as per manufacturer's protocol. Briefly, the culture medium from the samples was removed and 500 ​μl of staining solution [solution A (2 ​μl) ​+ ​solution B (1 ​μl) in 1 ​ml of DPBS] was added onto the micropatterns. After incubation for 20min at 37 ​°C, the samples were imaged using fluorescent microscope. The total number of cells (red and green) and the number of live cells (green) were counted using ImageJ software. The cell viability (%) was quantified as the percentage ratio of the number of live cells by total number of cells. For quantification, three independent samples (five images per sample) were used and reported as mean ​± ​standard deviation (SD).

### 2.11 Immunostainings

For immunostaining, all samples were rinsed with DPBS and fixed in 4% formaldehyde solution for 30min, washed with DPBS, after which cell membranes were permeabilized in 0.1% Triton X-100 for 10min. Afterwards, the samples were washed with DPBS for three times and then blocked with 1% FBS for 45min. For actin cytoskeleton staining, the samples were incubated with Alexa Fluor-647 Phalloidin (1:40 in DPBS) for 1hr at room temperature, followed by 10 ​min ​s incubation with Hoechst 33,342 (1:2000 in DPBS). For immunostainings, post blocking solution, the samples were washed with DPBS and incubated for overnight at 4 ​°C with CD31 monoclonal antibody (mouse, 1:200 in DPBS). The samples were then washed with DPBS and supplemented with Alexa Fluor 488 anti-mouse secondary antibody (1:1000 in DPBS) solution in dark. For double staining samples with F-actin and CD31, after 1hr incubation, Phalloidin 647 (1:40 in DPBS) was added into the secondary antibody staining solution and allowed to incubate for 1hr in dark. Thereafter, the samples were washed and Hoechst 33,342 (1:2000 in DPBS) was added for 10 ​min ​s in dark. Once stained, samples were washed, supplemented with DPBS (1 ​ml) and stored at 4 ​°C until imaged.

### 2.12 Image analysis

Image analysis was performed using maximum projection of confocal Z-stacks to reveal the filamentous actin and CD31^+^ endothelial cells network within both regions (aptamer & GelMA) of the micropattern. The orientation quantification was performed using maximum projection of confocal z-stacks, where images were processed via thresholding, binary and morphological segmentation tools in ImageJ software. The cytoskeletal actin stained cells and CD31^+^ endothelial cells network orientation was quantified using the built-in functions of ImageJ, where more than 100 ​cells (or CD31^+^ vessel networks) were selected from each segmented image (within each region) to best fit an eclipse with primary and secondary axis. The cell angle (0°-180°) was calculated as the angle between the primary axis and a line parallel to the *x*-axis of the image. The orientation data was represented as polar plot where “θ” and “n” corresponds to cell orientation and frequency counts binned in 15° or 20° increments, respectively. Similarly, images were processed and segmented to quantify the CD31^+^ endothelial vessel network properties such as vessel density, branching density, and average vessel length using Angiotool software. Herein, the vessel density is defined as the percentage of area occupied by CD31^+^ vessels within total image area (i.e., %vessels/total area), branching density as the number of vessel junctions/area and average vessel length as the mean length of all the vessels in image. All quantifications were performed with three technical replicates.

### 2.13 Statistical analysis

The statistical significance was determined by an independent Student *t-test* for two groups of data or two-way analysis of variance (ANOVA) with Tukey's multiple comparisons test using GraphPad Prism software. Data were calculated as mean ​± ​SD for replicates and p-values were presented as statistically significant with p ​< ​0.05.

## 3 Results and discussion

### 3.1 Patterned aptamer region display high design fidelity, aptamer retention, and CS recognition

To harness the unique structural and functional properties of aptamer-tethered growth factors for modulating vascular network morphogenesis and remodeling, we designed bicomponent micropatterns using aptamer-functionalized GelMA hydrogel (hereafter referred as ‘aptamer region’) and pristine GelMA hydrogel (hereafter referred as ‘GelMA region’) via free-radical polymerization initiated by visible light-based Ru/SPS photoinitiators. Compared to UV based photoinitiators, the Ru/SPS provides absorptivity in visible light range, high penetration depth and high molar absorptivity that can facilitate efficient curing at low initiator concentrations [[21], [22], [23]]. Two-step photolithography process was used to form spatially patterned aptamer and GelMA regions with defined specifications. DNA based oligonucleotide sequence specific for VEGF_165_ was utilized with 5′-acrydite modification that upon free-radical polymerization facilitates covalent incorporation of aptamers via the unsaturated bonds of acrydite group within GelMA polymeric network (Fig. 1). It has been previously demonstrated that 5′-acrydite modification of aptamer results in superior aptamer retention within bulk GelMA hydrogels as compared to non-modified aptamers, for up to 10 ​d at physiological temperature (37 ​°C). The polymeric networks with covalently linked aptamers elicit localized effects upon aptamer-biomolecule hybridization and limits its free diffusion within 3D matrices [13].

**Fig. 2:**
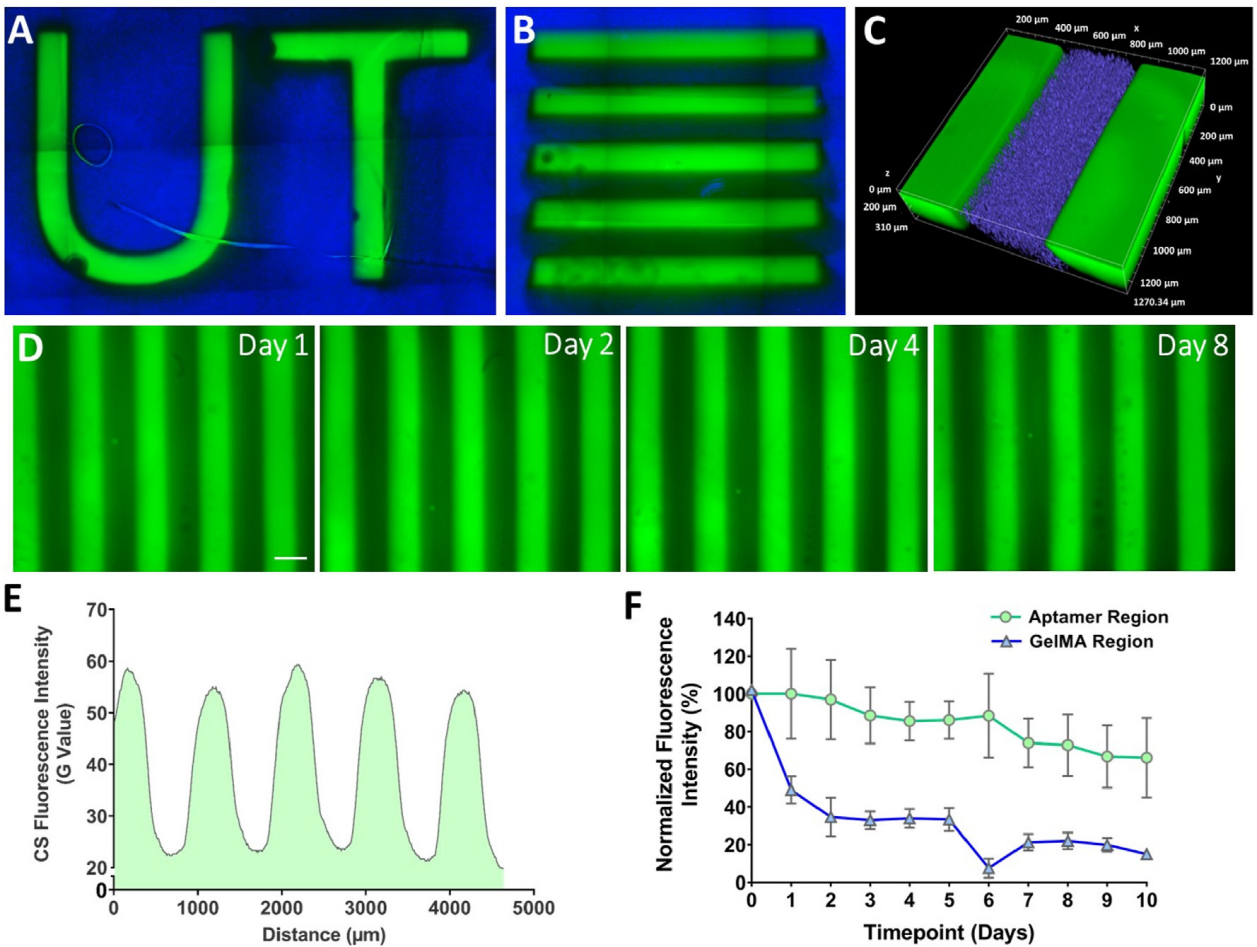
**Aptamer retention and molecular recognition of aptamer-CS**_**F**_**within micropatterns.** The stitched microscopic image of the (A) UT logo consisting of patterned aptamer region (line width ​= ​500 ​μm), overlaid with GelMA region (blue), (B) A_2_G_1_ micropattern with defined aptamer (green ​= ​500 ​μm) & GelMA regions (blue ​= ​500 ​μm), and (C) 3D projection of confocal Z-stacks (*z* ​= ​310 ​μm) of the A_2_G_1_ micropattern after 24hr CS_F_ incubation, confirming homogeneous CS_F_ retention in z-direction. The GelMA region with blue fluorescent microbeads (2 ​μm diameter) highlights distinct interface between both regions. (D) Representative fluorescence microscopic images of A_2_G_1_ micropattern after 24hr incubation with CS_F_ at different time-points. (E) The CS_F_ fluorescence intensity profile of A_2_G_1_ micropattern as a function of distance where green shaded region represents CS_F_ fluorescence intensity within micropattern across its width displaying high intensity peaks at regular intervals of 500 ​μm. (F) The normalized fluorescence intensity (%) of CS_F_ within aptamer and GelMA regions of micropattern till d10. The experiment was performed with n ​= ​3 experimental replicates (each sample selected for 10 ROIs within both regions) and values are represented as mean ​± ​SD.

The versatility of the aptamer-functionalized hydrogels in sequestering and controlling the release of target biomolecules (e.g., VEGF) is primarily determined by the molecular recognition among the target molecule, the aptamer, and the triggering CS. Therefore, to verify the aptamer's retention and the ability to sequester CS from the culture medium into the hydrogel matrix, we utilized 5′-Alexa Fluor 488 fluorophore labelled complementary sequence (CS_F_) at physiological temperature (i.e., 37 ​°C) (Fig. 2). Two-step photolithography process as explained in Fig. 1B, was employed for fabricating UT logo and A_2_G_1_ (5 ​× ​5 mm) micropatterns having patterned aptamer region overlaid with GelMA regions (Fig. 2A and B). The blue fluorescent microbeads embedded within patterned GelMA region was used to identify clear interfaces between aptamer and GelMA regions. Post CS_F_ incubation, patterned aptamer regions exhibited maximum fluorescence throughout the study duration (d10), validating stable aptamer incorporation and aptamer-CS_F_ molecular recognition within the hydrogel matrix. On the contrary, the patterned GelMA regions showed minimal detectable fluorescence after 48hr (Fig. 2D,F). A horizontal line intensity profile plot within A_2_G_1_ micropattern displayed intensity peaks and valleys consistent with parallel line widths of patterned aptamer region (Fig. 2F). The 3D projection of confocal Z-stacks confirm the homogenous sequestration of CS_F_ (z ​= ​310 ​μm) throughout the patterned aptamer region thickness while maintaining a clear interface with GelMA region (in blue color) (Fig. 2C and Video S1, Supporting Information**)**. Altogether, these observations validate aptamer's ability to sequestrate the CS_F_ into the polymer matrix at the macroscopic scale within 24hr incubation and showed functional molecular recognition properties via aptamer-CS_F_ hybridization at physiological temperature. The presence of clear interface between both regions with no signs of aptamer/CS_F_ leakage through other region proofs high specificity and affinity of CS_F_ towards the VEGF specific acrydite-modified aptamers.

The following is the supplementary data related to this article:

Multimedia component 2 Multimedia component 2

### 3.2 Presence of aptamer influences the local mechanical behavior of patterned regions

**Fig. 3:**
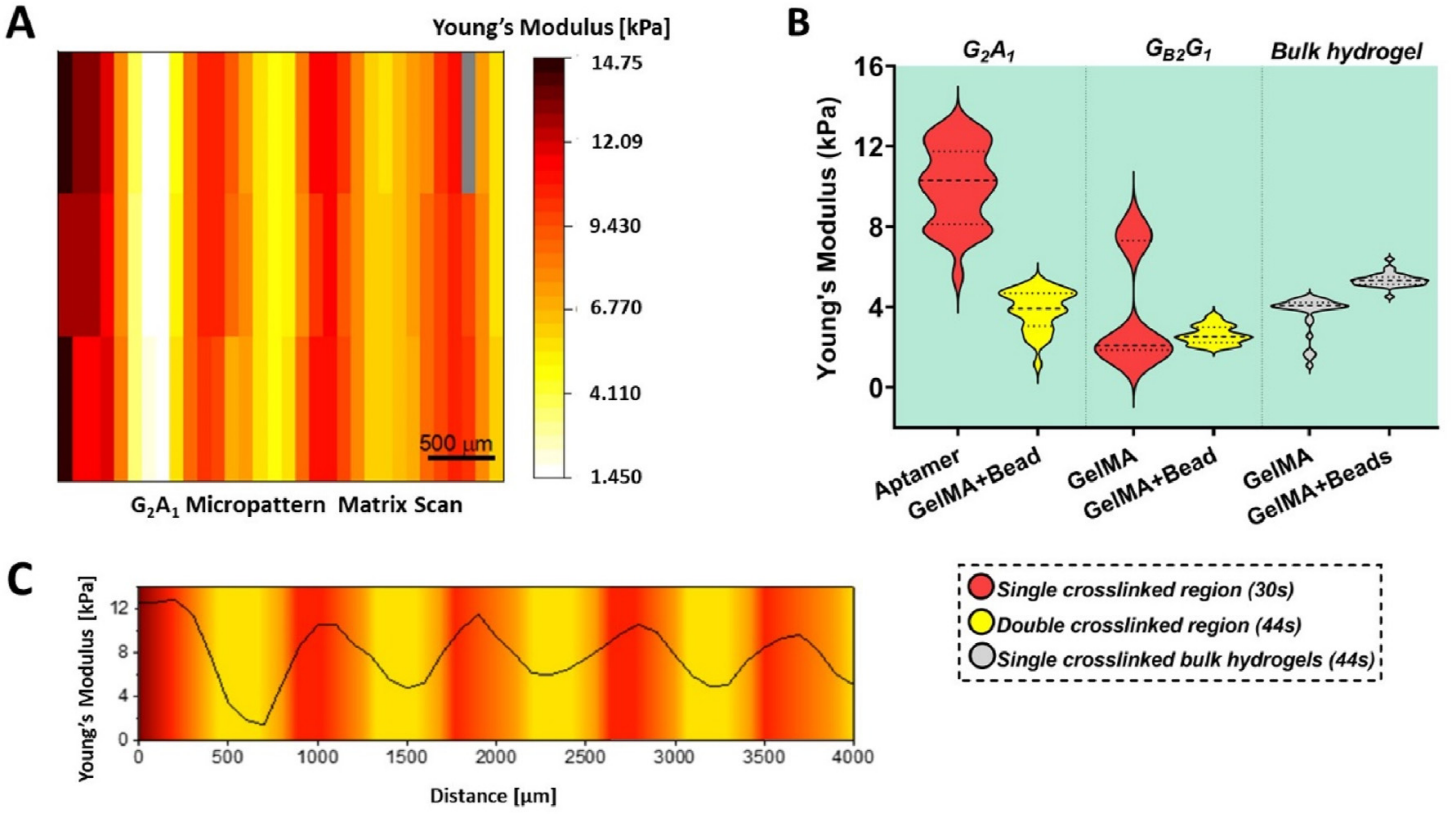
**Micromechanical behavior of bicomponent micropatterns as measured by nanoindentation at near physiological conditions.** (A) Matrix scan of G_2_A_1_ micropattern (aptamer line-500 μm; GelMA line-500 μm) for Young's modulus confirms difference in modulus among aptamer and GelMA regions. (B) Young's modulus of both regions (aptamer/GelMA) within G_2_A_1_, G_B2_G_1_ micropatterns and bulk GelMA/GelMA mixed with fluorescent microbeads hydrogels photocrosslinked for 44s. Red color violin plots corresponds to single photocrosslinked region (30s), yellow color for double photocrosslinked region (44s), during the two-step photocrosslinking process and gray color denotes bulk hydrogels continuously crosslinked for 44s. (C) Young's modulus profile of G_2_A_1_ micropattern as a function of distance perpendicular to the long axis of the pattern. The peak and trough of the modulus indicates the gradual changes between high modulus (red) aptamer region and low modulus (yellow) GelMA region in a gradient manner.

Having verified the aptamer incorporation and its stable CS_F_ hybridization within aptamer regions of the micropattern, we next set out to assess the effect of aptamer incorporation on micropattern's micromechanical behavior (Fig. 3). As mentioned, the process of two-step photolithography could lead to micropatterned regions with different crosslinking densities due to the variation in photocrosslinking time. For instance, in A_2_G_1_ micropatterns, the aptamer region is double crosslinked (14s ​+ ​30s ​= ​44s) compared to single crosslinked GelMA region (30s). The crosslinking density that is defined by the number of chemical or physical crosslinks present in a given volume controls fundamental hydrogel properties such as the volumetric swelling ratio (the volume ratio of water-swollen hydrogel to its dry polymer), the mechanical behavior and the diffusion coefficient of entrapped molecules, in an interdependent fashion [24]. A higher crosslinking density decreases the mesh size (which is a measure of available space between macromolecular chains for the diffusion), due to which an increase in mechanical properties such as shear modulus is expected [24]. These changes in hydrogel's fundamental properties could overall modulate the encapsulated cell behavior and influence the vascular network formation. To this end, the micromechanical properties of the micropattern were analyzed using arrayed nanoindentation at 37 ​°C. The matrix scan of G_2_A_1_ micropattern revealed differences in Young's modulus throughout the micropattern with high modulus patterned aptamer region (red) following low modulus patterned GelMA region (yellow/white) consecutively (Fig. 3A). The nanoindentation analysis corroborates with previous study with high average Young's modulus (10.06 ​± ​1.94 ​kPa) of single crosslinked patterned aptamer region (30s, red) compared to low modulus (3.87 ​± ​0.99 ​kPa) within double crosslinked patterned GelMA region (with blue microbeads) (44s, yellow) of the same G_2_A_1_ micropattern (Fig. 3B). Although the exact mechanism remains unclear, the observed discrepancy among the modulus of different patterned regions is likely attributable to the covalent incorporation of aptamers within GelMA matrix that in-turn could affect the crosslinking density and mechanical modulus of the hydrogel [13]. Notably, aptamers (when present in higher amounts) are hydrophilic in nature which increases swelling of the hydrogel leading to higher Young's modulus and larger pore sizes. SEM imaging of aptamer-functionalized hydrogels with same aptamer concentration (25 ​μM) revealed larger pore sizes than the GelMA hydrogels at same polymeric concentration [13]. Even though SEM images do not directly reveal the interchain distances due to possible packing of polymer chains during the lyophilization process, the observed pore sizes provides an indicative measure of the void fractions. The pore size and mesh size (directly indicative of interchain distance) have a positive correlation. Furthermore, no significant difference in Young's modulus was observed among single crosslinked plain GelMA region (30s; 3.81 ​± ​2.72 ​kPa) and double crosslinked GelMA with microbeads region (44s; 2.61 ​± ​0.45 ​kPa) of the control G_B2_B_1_ micropattern, suggesting negligible effect of microbeads within patterned GelMA regions in modulating their mechanical behavior (Fig. 3B). To understand the influence of double crosslinking on the micropatterns, bulk hydrogels photocrosslinked till 44s continuously were also analyzed. The bulk GelMA and GelMA with microbeads hydrogel showed the average Young's modulus of 3.66 ​± ​0.94 ​kPa and 5.35 ​± ​0.33 ​kPa, respectively (Fig. 3B). As expected, the bulk plain GelMA and patterned GelMA region within micropatterns showed modulus within same range, thus negating an effect of double photocrosslinking on the mechanical properties. The higher modulus of bulk GelMA with microbeads hydrogels can be understood as an aberration caused due to proximity of the nanoindentation tip to the stiffer polystyrene microbeads embedded within GelMA matrix during the measurements. Interestingly, the matrix scan as well as the Young's modulus profile as a function of distance perpendicular to the long axis of G_2_A_1_ micropattern (Fig. 2A,C) revealed gradual modulus (stiffness) changes after every 500 ​μm, as opposed to sharp modulus differences. This observation may be attributed to a bleed through effect (scattering) of light through the photomask and diffusion of free radicals into the surrounding areas. It is also worth noting that the two-step photocrosslinking process involves overlaying the second pre-polymer onto the first crosslinked pattern, which could lead to the formation of thin film of second pre-polymer over the entire micropattern surface, thus contributing towards the gradual change of stiffness in both regions. Altogether, these results establish the difference in Young's modulus among both regions (aptamer and GelMA) of the bicomponent micropattern in a gradient manner.

### 3.3 Aptamer-tethered VEGF localization within micropatterns

**Fig. 4:**
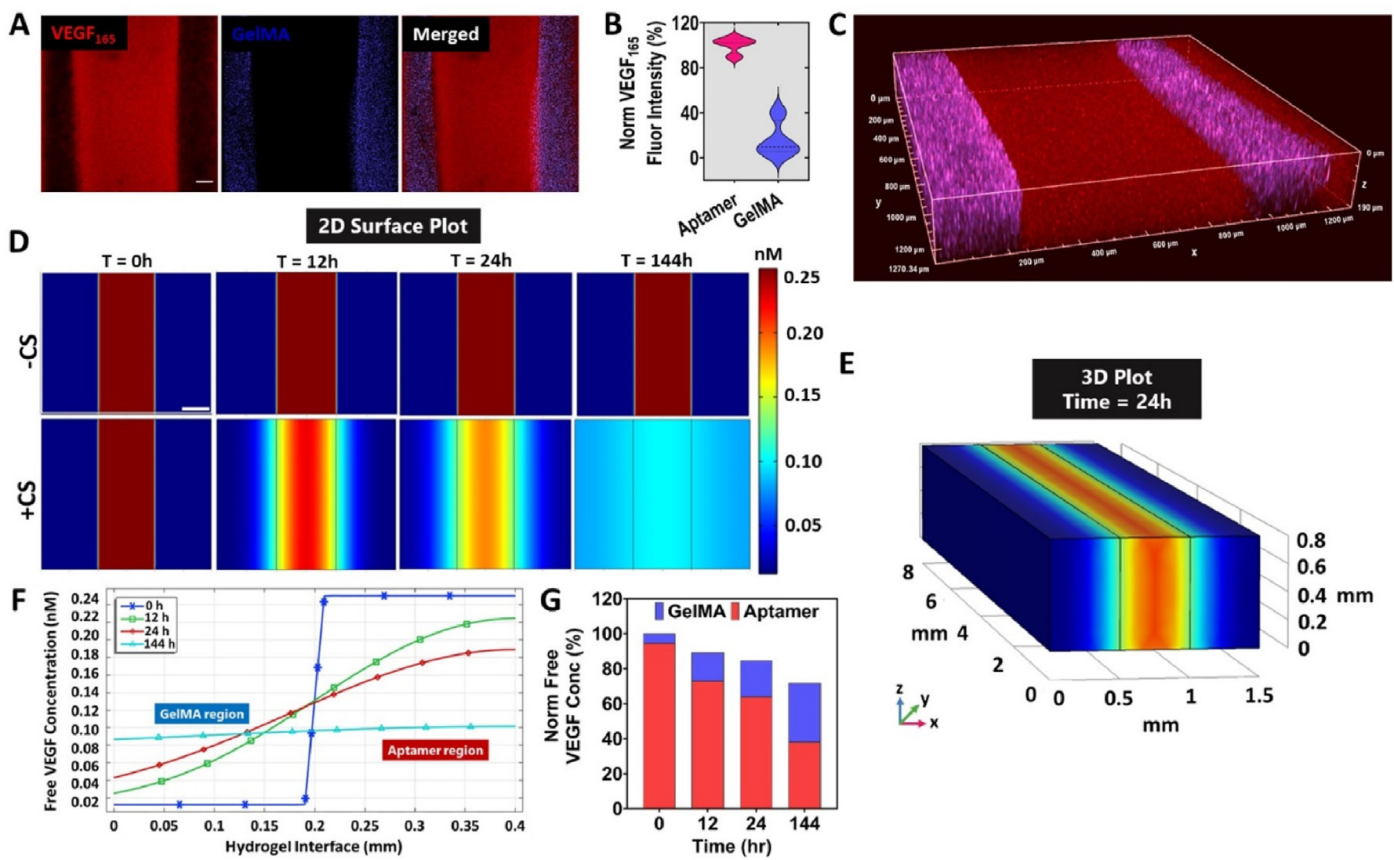
**Aptamer-tethered VEGF localization within micropatterns.** (A) Maximum projection confocal Z-stacks montage showcasing A_2_G_1_ micropattern immunostained for VEGF_165_ (red color, 800 ​μm) confirms maximum VEGF sequestration within aptamer region. The blue color within neighboring GelMA regions (500 ​μm) corresponds to the fluorescent microbeads mixed in GelMA pre-polymer. The experiment was performed with n ​= ​2 experimental replicates and scale bar is 100 ​μm. (B) Normalized fluorescence intensity (%) of VEGF within aptamer and GelMA regions of A_2_G_1_ micropattern. (C) 3D projection of confocal Z-stacks of VEGF immunostained A_2_G_1_ micropattern confirms homogenous VEGF sequestration throughout the micropattern thickness (z ​= ​190 ​μm). *In silico* analysis of free VEGF transport among aptamer and GelMA regions of the micropattern using reaction-diffusion model in COMSOL Multiphysics software. (D) The 2D surface plot and (E) 3D volume plot of free VEGF diffusion from the high VEGF concentration region (aptamer) to low concentration regions (GelMA) upon triggered release in the presence (or absence) of CS over time. The scale bar in (D) represents 200 ​μm. (F) The free VEGF concentration graph as a function of distance across the hydrogel interface showing the change in VEGF concentration in both regions over time. The simulation data confirms maximum VEGF concentration within aptamer region at the start of free diffusion that changes to 0.5 ​μM from 0.8 ​μM within 24hr of CS addition into the culture medium which stabilizes over time with an equilibrium by 144hr (d6). (G) Normalized free VEGF concentration % graph displaying maximum free VEGF in aptamer region at 0hr that equalizes by 144hr among both regions.

Having established the difference in micromechanical behavior among both regions of bicomponent micropattern, we next aimed to examine the versatility of patterned aptamer region for VEGF sequestration from the medium and its localized retention within micropatterns (Fig. 4). To this end, A_2_G_1_ micropatterns with patterned aptamer region (800 ​μm) and GelMA region (500 ​μm) were fabricated, followed by VEGF (10 ​ng) incubation for 1hr. The qualitative and quantitative confocal image analysis confirmed higher VEGF sequestration (in red color) localized within aptamer region, as compared to GelMA region (in blue color) of the micropattern within 1hr (Fig. 4A and B). In accordance with the microscopic images, the VEGF fluorescence intensity normalized with aptamer region showed only 16% intensity in the GelMA region of the micropattern. Furthermore, the 3D projection of confocal Z-stacks reveal homogenous distribution of immunostained VEGF throughout the depth (z ​= ​190 ​μm) of aptamer region (Fig. 4C). These results corroborate with literature, where higher VEGF amounts (46%) were sequestered within bulk aptamer-functionalized hydrogels compared to bulk GelMA hydrogels (33%) within 1hr of incubation [13]. As indicated in Fig. 4A, the GelMA regions show minimal VEGF presence (∼15% of VEGF fluorescence intensity) in contrast to the previously reported 33% within bulk GelMA hydrogels [13]. It is speculated that the presence of patterned aptamer and GelMA regions within the same micropattern may induce competitive VEGF sequestration where aptamer's higher affinity towards VEGF molecules cause aptamer-VEGF hybridization, overriding the gelatin's inherent electrostatic interaction. This competitive VEGF sequestration behavior could thus be attributed to rapid and localized high VEGF sequestration confined within aptamer regions after 1hr of VEGF loading.

Aptamers can be selected from oligonucleotide libraries to specifically bind to any target biomolecule (e.g., VEGF) with high affinity [25,26]. However, aptamers exhibit reversible interaction with these target biomolecules in the presence of CS. Due to this aptamers can not only bind the target molecules with high specificity and affinity, but can also release it on-demand by dissociating the aptamer-biomolecule complex using CS as an external trigger. We have previously reported that by using 1:1 ​mol ratio of aptamer to CS, 20–25 ​pg VEGF can be triggered to release within 24hr and cumulative release of 30 ​pg over 10 ​d can be achieved from the bulk aptamer-functionalized hydrogels [13]. On the contrary, the bulk GelMA hydrogel showed burst VEGF release (∼19 ​pg) within initial 24hr after loading, followed by negligible VEGF release throughout the study duration or after triggered release, making up to 25 ​pg cumulative release over 10 ​d [13]. We speculate that similar on-demand VEGF release profiles could be achieved from aptamer region of the micropattern upon CS triggering, while maintaining the same aptamer to CS mole ratio (1:1) [13]. To investigate the effect of CS triggering on micropatterns and the diffusion profiles of released free VEGF through the micropattern regions, we next explored reaction-diffusion based simulations using COMSOL Multiphysics software.

Numerical simulations were carried out to map the spatiotemporal concentration profiles of the released free VEGF from aptamer region to the adjacent GelMA regions after CS addition into the medium (0 ​h) till d6 (144 ​h). This timeline was chosen to mimic the experimental timeline of cell-laden bicomponent micropattern with CS addition on d4 and samples being cultured till d10. Assuming negligible VEGF release in the absence of CS, we considered the VEGF diffusivity in the non-treated micropatterns to be zero. This assumption is in accordance with literature where ELISA data confirmed near-zero VEGF release from the bulk aptamer-functionalized hydrogels without CS trigger [13]. Generally, diffusion is the prime mechanism controlling release of growth factors from hydrogel. However, within aptamer-functionalized hydrogel, the release of growth factors is determined by the combinatorial effect of diffusion and the reaction-binding kinetics between aptamer and growth factor. Considering this, the apparent free VEGF diffusivity within aptamer-functionalized hydrogels has been reported to be (18 ​± ​2.2)×10^−10^ ​cm^2^/s [27]. The 2D simulation data indicates high amounts of free VEGF release upon CS addition at 0 ​h from the aptamer regions (0.247 ​nM) that soon starts to gradually diffuse out into the surrounding GelMA regions (0.013 ​nM) (Fig. 4D,E,F and Videos S2 and S3). The diffusion creates an instantaneous gradient of free VEGF concentration with aptamer regions displaying higher concentrations (0.167 ​nM) than GelMA region (0.053 ​nM) after 24hr (Fig. 4F and G). The 3D simulations after 24hr displays presence of homogeneous VEGF concentration gradients throughout the interface between aptamer and GelMA regions (Fig. 4E). The established VEGF concentration gradient after 24hr with high VEGF concentration in aptamer regions (64.02%) than GelMA regions (20.65%) soon reaches an equilibrium after 144hr where both aptamer as well as GelMA regions showed similar VEGF concentrations 38.23% and 33.55%, respectively (Fig. 4F and G and Video S3). The micropatterns without CS treatment showed no free VEGF release, as assumed in the model. In hydrogels, many factors such as pore size affects the diffusivity of growth factors within its polymeric matrix. For instance, larger pore sizes results into higher apparent diffusivity of growth factors. On the contrary, the apparent diffusivity within aptamer-functionalized hydrogels is controlled by the density and binding affinity of aptamers, rather than its pore size [27]. Therefore, it is possible to modulate the VEGF release profiles that affects their bioavailability to elicit specific cell responses, by tuning both the binding affinity and density of aptamers within aptamer-functionalized hydrogel [28]. Taken together, these results confirm the formation of instantaneous VEGF gradients throughout the micropattern upon CS treatment within the initial 24hr, thus highlighting the precise control of VEGF bioavailability within micropattern regions in both space and time.

The following are the supplementary data related to this article:

Multimedia component 3 Multimedia component 3

Multimedia component 4 Multimedia component 4

### 3.4 Bioactivity of cell-laden aptamer-tethered VEGF micropatterns and its triggered release via CS hybridization

**Fig. 5:**
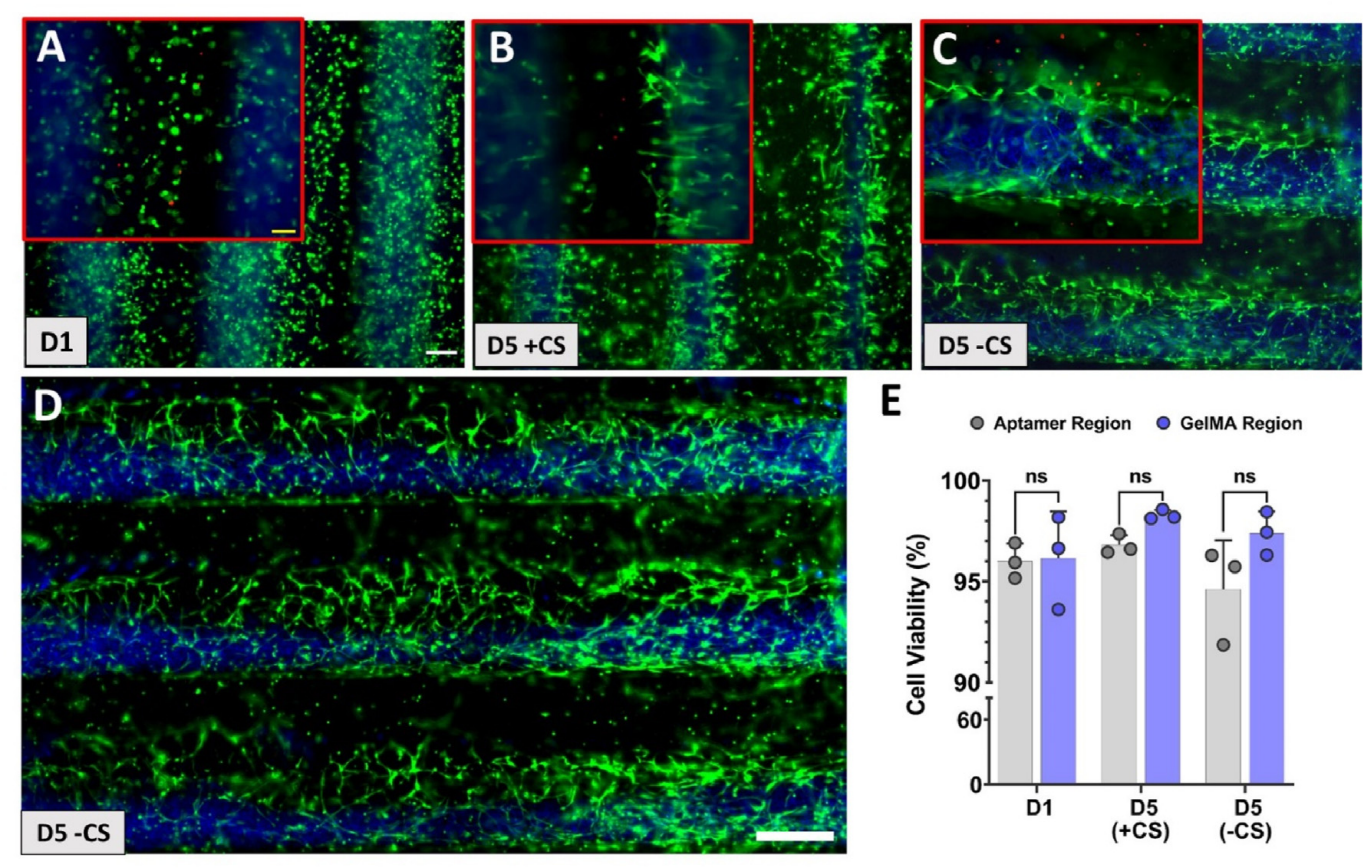
**Cell viability of 3D co-cultured cell-laden micropattern in the presence or absence of triggered VEGF release.** (A) Live/Dead stained fluorescent microscopic images of HUVECs/MSCs co-cultured A_2_G_1_ micropatterns (aptamer line- 500 ​μm; GelMA line, blue- 500 ​μm) with VEGF_165_ specific aptamer-functionalized hydrogel in the presence or absence of CS addition on d4. The microscopic images of micropattern at 4× magnification and the inset shows zoomed representative image at 10× magnification of samples on (A) d1. For comparison, (B) d5 samples with triggered VEGF release due to CS addition into the co-culture medium on d4 (D5 +CS) were compared with (C) samples without CS addition on d4 (D5 –CS). The scale bar (white) is 200 ​μm and inset scale bar (yellow) is 100 ​μm. (D) Representative stitched microscopic image (at 10× magnification) of sample with triggered VEGF release on d5 (D5 +CS). The green and red color represents live and dead cells, respectively. Blue color corresponds to the GelMA region mixed with blue fluorescent microbeads in the micropattern. Scale bar is 500 ​μm. (E) Cell viability % of micropattern on d1, d5 w/CS and w/o CS quantified using ImageJ software with three experimental replicates, n ​= ​3. The statistical significance was calculated using two-way ANOVA with Tukey's multiple comparisons test where alpha was fixed at 0.05 with ∗∗∗p ​= ​0.0005, ∗∗∗∗p ​< ​0.0001 and ns stands for not significant.

Having established the aptamer-CS molecular recognition and stable VEGF sequestration ability of the patterned aptamer regions, we next evaluated the biocompatibility of the micropatterns throughout the process of photopatterning, VEGF loading and CS mediated release, along with the bioactivity of the aptamer-tethered VEGF (Fig. 5). Fluorescence microscopic images of HUVECs/MSCs 3D co-cultured micropatterns confirmed homogeneously distributed cells that exhibit round morphology on d1 and elongated into spindle-like morphology by d5 (Fig. S2). As indicated in Fig. 5, high cell viability (>95%) was observed among both regions (aptamer region-500 μm; GelMA region-500 μm) of the A_2_G_1_ micropattern on d1. Insignificant differences in cell viability were observed upon CS triggering among both patterned regions on d5 (aptamer region: 96% with CS & 94% without CS; GelMA region: >97% in with & without CS), but high viability across the micropattern regions was observed at all time-points, irrespective of CS triggering (Fig. 5E). On d5, the cells encapsulated within patterned aptamer regions (irrespective of CS treatment) displayed round morphology with minimal cell spreading compared to GelMA region (Fig. 5B,C,D), which could potentially be attributed to the disparity in stiffness among the two regions, as confirmed by nanoindentation measurements (Fig. 3). The Ru/SPS photo-initiating system generates sulfate radicals upon visible light (400–450 ​nm) induced photo-initiation, that in-turn can react with the unsaturated carbon moieties present within GelMA backbone and acrydite-modified aptamers, to form covalent bonds. Compared to UV photoinitiators, the Ru/SPS provides better penetration depth and cytocompatibility, but an increased radical generation due to Ru/SPS photo-initiation might induce oxidative damage to the encapsulated cells [21,29]. The observed enhanced biocompatibility in our study could be attributed to the combinatorial effect of gelatin's free radical scavenging properties [30,31], presence of cell attachment (RGD) as well as matrix remodeling (MMP-degradable) functional amino acid motifs within GelMA [32], and high bioactivity of the aptamer-tethered VEGF molecules within the micropattern.

### 3.5 Spatially patterned aptamer-tethered VEGF affects cellular orientation

**Fig. 6:**
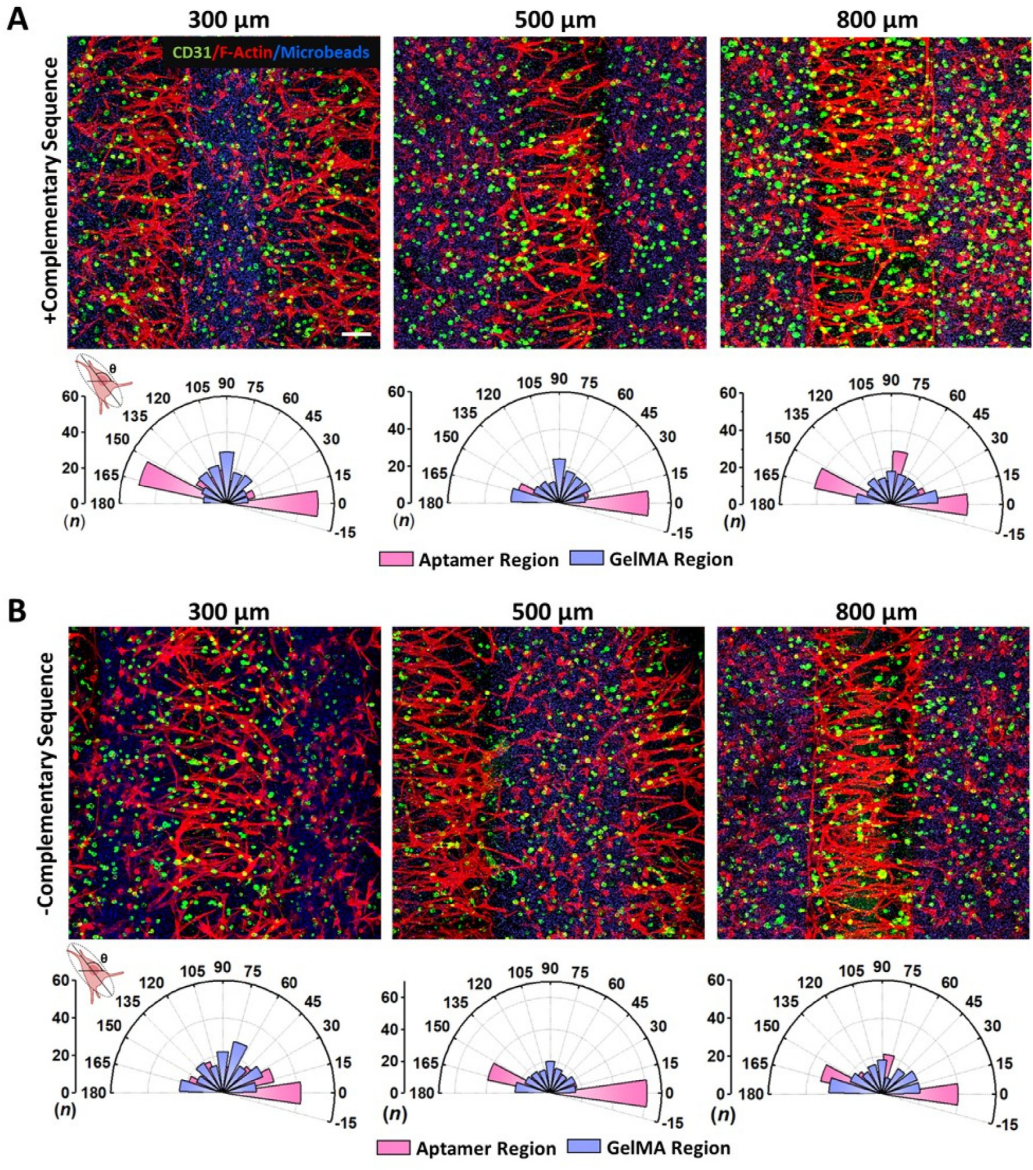
**Patterned aptamer-tethered VEGF elicits spatially defined cellular alignment within micropatterns.** Maximum projection of confocal Z-stack image showing F-actin (red) and CD31 (green) stained G_2_A_1_ micropatterns on d5 that were (A) treated with CS to trigger the VEGF release on d4 and (B) no CS treatment. The samples were quantified for individual cell orientation within aptamer and GelMA regions of the micropattern using F-actin stained microscopic images. The data is represented as polar plot where “θ” & “n” represents cell orientation angle and frequency counts binned in 15° increments, respectively. The quantification was performed with three technical replicates, n ​= ​3. The blue color corresponds to fluorescent microbeads present in GelMA region. For comparison, three micropattern designs were studied that have fixed aptamer region (500 ​μm) but varies in GelMA region (300/500/800 ​μm), respectively. The scale bar represents 100 ​μm.

The microscopic images of cells within A_2_G_1_ micropattern revealed a difference in cellular orientation among the CS treated and non-treated micropatterns on d5 (Fig. 5B and C). Specifically, the CS treated samples displayed cells aligned perpendicular to the direction of the micropattern's long axis (Fig. 5B), compared to non-treated samples where cells were wrapped around the pattern (Fig. 5C). The observation could be ascribed to various reasons including the photopatterning procedure or VEGF localization. To understand this behavior, we next evaluated micropatterns with reversed order of photopatterning i.e., double crosslinked GelMA region and single crosslinked aptamer region (G_2_A_1_). If only high stiffness among double crosslinked aptamer regions was hampering the cell spreading and proliferation, the G_2_A_1_ micropatterns should elucidate higher cellular responses within single crosslinked aptamer region. Additionally, to understand the extent of localized aptamer-tethered VEGF for eliciting specific cell responses, aptamer regions with spatially defined GelMA spacings (without VEGF localization) within micropatterns can be proven as a guiding tool for collective cell behavior. To this end, the cell-laden bicomponent micropatterns with constant aptamer region (500 ​μm) for VEGF localization amid different GelMA spacings (300/500/800 ​μm) were studied with/without CS treatment. Confocal microscopy was used to assess 3D actin cytoskeleton organization and indicated increased cell adherence, spreading and sprouting of encapsulated cells within the aptamer region compared to GelMA region on as early as d3 of culture within all micropattern designs (Fig. S3). Notably, on d3 of culture major cell population in aptamer region reorganized perpendicular to the direction of the long axis of micropatterns, almost bridging the two regions together in all the designs (Fig. S3). For instance, aptamer region in 300 ​μm design showed high cell frequency among 160°-180°(n ​= ​43) and 80°-100°(n ​= ​35) cell orientations, whereas the 500 ​μm and 800 ​μm designs showed majority of cell populations to be perpendicularly aligned to the pattern's long axis [500 ​μm:0°-20°(n ​= ​43),160°-180°(n ​= ​39); 800 ​μm:160°-180°(n ​= ​37)]. On the contrary, GelMA regions displayed higher cell alignment in 300 ​μm designs [0°-20°(n ​= ​40),80°-100°(n ​= ​36),100°-120°(n ​= ​30),120°-140°(n ​= ​28)] than 500 ​μm and 800 ​μm designs, where cells were mostly randomly oriented (Fig. S3). This effect became more profound by d5 and similar trends were observed where cells encapsulated within aptamer regions displayed highly aligned cell spreading and sprouting in all the designs compared to GelMA regions. The CD31^+^ stained confocal images revealed round morphology of endothelial cells in all the samples on d5 (Fig. 6).

Endothelial sprout orientation during sprouting morphogenesis within hydrogels has been shown to be regulated by VEGF gradients [33,34]. Spatially patterned aptamer regions competitively sequester VEGF, making it locally available in high VEGF concentration. On the other hand, GelMA regions being dependent on electrostatic interaction, are only able to retain minimal VEGF concentration, as confirmed by anti-VEGF immunostaining and COMSOL simulations (Fig. 4). This disparity in VEGF concentration among both regions is speculated to create spatially defined VEGF concentration gradient at the interface. As shown in Fig. 3A,C, the stiffness gradient is observed among the patterned regions of micropattern that relates to the light deflection during photopatterning, where light passes through the photomask at a titled angle instead of a straight line, and thus leading to photopatterned regions overlapping at titled angle throughout its depth (Fig. S4). We speculate that the observed unique cellular directionality confined within patterned aptamer regions can possibly be linked to the aptamer-tethered VEGF localization in gradient manner due to the tilted overlapping of both patterned regions. Besides, in CS-treated samples, corroborating with simulation results, the aptamer-CS hybridization facilitates disassociation of aptamer-VEGF complex enabling diffusion of released free VEGF from the aptamer region to adjacent GelMA regions in a gradient manner within initial 24hr of CS treatment (Fig. 4E). Evidently, high cellular alignment along the direction of VEGF gradient in all micropattern designs on d5 confirms that the aptamer-tethered VEGF is not only bioactive but can also guide encapsulated cells to elicit localized responses such as adherence, spreading, sprouting and alignment within 3D microenvironment. As expected, the increase in GelMA spacings decreased cellular alignment within aptamer regions. For instance, the CS treated 300 ​μm spacings resulted in highest cell frequencies aligned perpendicular to the micropattern's long axis [0°-20°(n ​= ​51),160°-180°(n ​= ​50)], followed by lower cell frequencies in 500 ​μm [(0°-20°(n ​= ​48),160°-180°(n ​= ​23)] and 800 ​μm spacings [(0°-20°(n ​= ​41),80°-100°(n ​= ​29),160°-180°(n ​= ​42)] (Fig. 6A). However, patterned GelMA regions of CS treated micropatterns showed maximum cell frequencies aligned parallel to the micropattern's long axis with minimum spacing [300 ​μm: 80°-100°(n ​= ​29)]. As the GelMA spacings increased, the cellular alignment within patterned GelMA regions gradually changed from parallel to perpendicular direction of the long axis [500 ​μm: 80°-100°(n ​= ​24),160°-180°(n ​= ​26)] and with 800 ​μm spacing negligible directionality was observed where cells aligned homogeneously at all angles (Fig. 6A). It is speculated that the smaller GelMA spacing facilitates sharp VEGF concentration gradients at the interface compared to higher spacings that spreads out the gradient in larger spacings. Similar cellular alignment trends were observed in CS treated and non-treated samples, except within 300 ​μm designs, the non-treated samples exhibited higher cellular alignment specifically at 0°-20°(n ​= ​42) than the CS-treated samples, which could be due to the combinatorial effect of high VEGF localization (no CS trigger) with minimum spacings to elicit maximum cellular alignment (Fig. 6B). In non-treated micropatterns, the aptamer region of 500 ​μm samples displayed high cell frequencies aligned perpendicular [0°-20°(n ​= ​61),160°-180°(n ​= ​40)], whereas 800 ​μm samples showed high cellular frequencies aligned both perpendicular and parallel to the pattern's long axis [0°-20°(n ​= ​40),160°-180°(n ​= ​33),80°-100°(n ​= ​21)]. The patterned GelMA region of non-treated micropatterns exhibited cell frequencies aligned parallel in minimum spacings [300 ​μm: 60°-80°(n ​= ​28)] and perpendicular to pattern's major axis in maximum spacing samples [800 ​μm: 160°-180°(n ​= ​28)]. Lower cell frequencies aligned homogenously at all directions were observed in patterned GelMA regions of 500 ​μm samples (Fig. 6B). Besides, the patterned GelMA regions in all samples exhibited comparatively lower cellular responses and reduced directionality that could possibly be linked to the low VEGF localization within GelMA regions.

In all samples, the mechanically stiffer patterned aptamer regions elucidated higher cell response than the relatively softer patterned GelMA regions. Although aptamer functionalization within polymer matrix could increase the overall hydrogel's stiffness (Fig. 3), the aptamer-tethered VEGF localization was able to overcome its mechanical constraints and elicit spatially defined specific cell responses (Fig. 6). Furthermore, the cells in close proximity to the perimeter of both regions tended to align along the long axis of the micropatterns (Figs. S3 and 6). The parallel cellular alignment along the micropattern long axis corroborates with literature where the process of photopatterning have been shown to influence endothelial cells orientation *in vitro* [35]. The mechanical stresses generated via traction forces cause accumulation of higher stresses on the periphery of patterned features, followed by initiation of patterned proliferation that triggers tubular morphogenesis during angiogenesis [36].

### 3.6 Spatiotemporally controlled tubular microvascular network formation within patterned aptamer region

**Fig. 7:**
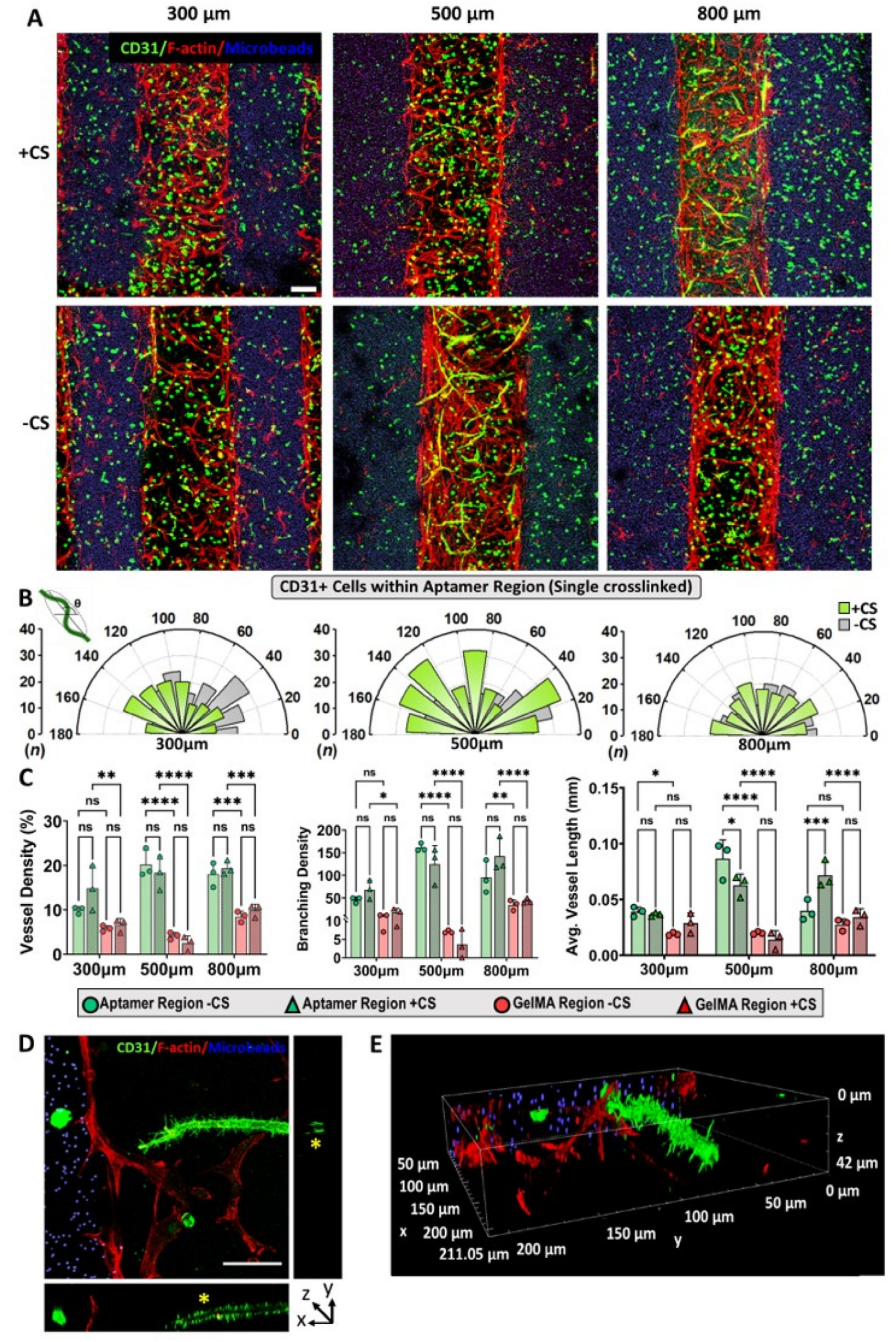
**Spatiotemporally patterned tubular microvascular network within patterned aptamer region of bicomponent cell-laden micropatterns.** (A) Maximum projection confocal *Z*-stack images showing cell cytoskeletal F-actin (red) and endothelial cells specific CD31 (green) expression within HUVECs/MSCs co-cultured, immunostained bicomponent G_2_A_1_ micropatterns on d10 (with or without CS treatment on d4). The blue color corresponds to fluorescent microbeads present within GelMA region. For comparison, three micropattern designs were studied that have fixed aptamer region (500 ​μm) but varies in GelMA region (300/500/800 ​μm), respectively. The scale bar is 100 ​μm. (B) Quantification of CD31 expressing endothelial cells network orientation within aptamer region of G_2_A_1_ micropatterns (300/500/800 ​μm). The polar plots represents “θ” & “n” as endothelial cells network orientation angle and frequency counts binned in 20° increments, respectively. The quantification was performed with three technical replicates, n ​= ​3. (C) CD31 expressing endothelial cells vessel network properties quantification within both regions of G_2_A_1_ micropattern (with or without CS treatment on d4) on d10. The values are represented as mean ​± ​SD, along with individual data points. The calculations were performed with three technical replicates, n ​= ​3. The statistical significance was calculated using two-way ANOVA with Tukey's multiple comparisons test where ∗p ​< ​0.05, ∗∗p ​< ​0.01, ∗∗∗p ​< ​0.001, ∗∗∗∗p ​< ​0.0001 and ns stands for not significant. (D) Orthogonal view and (E) 3D projection of the confocal Z-stack (stack thickness, z ​= ​42 ​μm) showing F-actin & CD31 immunostained micropattern at 60× magnification exhibiting the growing CD31^+^ microvascular structure at the interface between aptamer and GelMA (blue microbeads) regions. At the cross-section of the vessel, round lumen like vascular structure could be observed in orthogonal view (indicated by yellow ∗). The scale bar is 50 ​μm.

Controlling VEGF bioavailability within engineered matrices in both space and time is vital for directing tubular morphogenesis of endothelial cells self-assembly into mature and stable vascular networks. Having established the ability of aptamer-tethered VEGF in eliciting spatially defined cellular alignment within patterned regions, we next investigated its ability on directing tubular endothelial morphogenesis in a spatiotemporally controlled fashion. Upon studying the G_2_A_1_ micropatterns on d10, the samples displayed spatially confined high cellular responses (cell adherence, spreading, and sprouting) within patterned aptamer regions compared to patterned GelMA regions in all designs. Interestingly, by d10 no obvious cell alignment was observed in both patterned regions. As indicated in Fig. 7A, the co-cultured patterned aptamer regions exhibited CD31 positive spatially confined, stable microvascular network formation confirming tubular endothelial morphogenesis. Furthermore, high magnification (60x) microscopic images in orthogonal view revealed elliptical/circular lumen-like structure (marked with yellow asterisk∗) covered with microfilaments filopodia-like structures on CD31^+^ microvascular networks (Fig. 7D and E). The 3D projection confirms its tubular morphology distributed homogenously through the depth of the hydrogel Z-stack (z ​= ​42 ​μm). As shown in Fig. 7D, the filopodia-like structures covering the tubular network's surface shows spreading in all directions and could be speculated to participate in sensing its surrounding microenvironment as well as locating other cells within the hydrogel in 3D **(**Video S4**)**. It should be noted that observed lumens accounts for 5–10 ​μm diameter, suggesting that the luminal structures are made up by either one or two endothelial cells folding together into lumen shape. Actin is a highly dynamic and mechanosensitive cytoskeletal component in endothelial cells, that undergoes constant remodeling into filopodia, lamellipodia and stress fibers for facilitating cell migration during angiogenesis [37]. Previous studies have shown a difference of 1.5 fold in actin production among encapsulated HUVECs within stiffer hydrogels compared to relatively softer ones [38]. Therefore, we speculate that due to the combinatorial effect of softer hydrogel matrices as well as the continuous cytoskeleton remodeling due to VEGF mediated chemotactic cell migration resulted in the lack of visible F-actin positive staining within the microvascular networks. However, the presence of filopodia on the surface of microvascular networks confirms the functional actin cytoskeletal machinery within these luminal endothelial cells.

The following is the supplementary data related to this article:

Multimedia component 5 Multimedia component 5

The quantification of CD31^+^ network alignment within patterned aptamer regions confirmed maximum influence of CS treatment in 500 ​μm spacing samples where majority of cells aligned at specific orientations [20°-40°(n ​= ​35),80°-100°(n ​= ​32),120°-140°(n ​= ​34)], whereas random distribution of cell orientation with majority cells aligned at ≤60° [(20°-40°(n ​= ​30),40°-60°(n ​= ​23),0°-20°(n ​= ​22)] was observed in non-treated samples (Fig. 7B). Upon comparing all CS treated and non-treated samples, the 300 ​μm and 800 ​μm spacings exhibited lower cell frequencies with random orientations compared to 500 ​μm spacing samples (Fig. S5). For instance, the CS treated 300 ​μm samples exhibited low cell frequencies with majority cells aligned between 100°-160° [(100°-120°(n ​= ​21),120°-140°(n ​= ​22),140°-160°(n ​= ​24)] and at ≤60° [(0°-20°(n ​= ​21),20°-40°(n ​= ​24),40°-60°(n ​= ​30)] in non-treated samples. These observations confirm 500 ​μm as the optimal spacing for influencing the orientation of developing endothelial network within patterned aptamer regions, where CS triggered VEGF release tended to amplify this effect. It is speculated that by d10 of culture, the VEGF gradient effect generated due to aptamer-CS hybridization tends to attain equilibrium, as indicated by COMSOL simulations (Fig. 4D & Video S3). These changes directly relate to the high degree of cells aligning perpendicular to the pattern's long axis on d5 compared to d10 results where majority of CD31^+^ cells/network tended to randomly align within aptamer regions.

Confocal microscopy images revealed that spatiotemporal control over VEGF bioavailability locally regulates tubular endothelial morphogenesis confined within patterned aptamer region. In correlation with network orientation results, the CD31^+^ endothelial network properties analysis within patterned aptamer region on d10 highlighted insignificant effect of CS treatment on network properties in most spacings (Fig. 7C). However, significantly higher average vessel lengths were observed in 500 ​μm designs without CS (0.086 ​± ​0.016 ​mm) than CS treated samples (0.062 ​± ​0.009 ​mm) and in 800 ​μm designs with CS treated (0.071 ​± ​0.012 ​mm) than non-treated (0.039 ​± ​0.009 ​mm). Similar trends were observed for other vessel properties such as vessel density (%) and branching density, despite their insignificant differences. Unlike other designs, the 500 ​μm design showed higher vessel density (20.23 ​± ​3.24% & 18.34 ​± ​3.81%) and branching density (161.36 ​± ​8.46 & 124.78 ​± ​40.94) in non-treated compared to the CS-treated samples, respectively. Among all designs, the 500 ​μm samples exhibited significantly higher vessel parameters compared to others in the following order: 300 ​< ​800<500 ​μm. As expected, the patterned GelMA regions displayed minimal network properties with no significant difference among CS treated and non-treated samples, in all designs. These trends corroborated with other vessel parameters such as vessel density% and branching density among CS-treated and non-treated GelMA region of all designs (Fig. 7C). Altogether, these results confirmed minimal vessel network formation in 300 ​μm designs, regardless of CS treatment, and establishes 500 ​μm as the optimal spacing for guiding the tubular endothelial morphogenesis. Specifically, non-treated 500 ​μm samples showed enhanced network formation compared to the CS-treated samples, thus highlighting the need of localized VEGF within non-treated 500 ​μm design (aptamer region) for directing the vessel network formation. Although comparatively lower than 500 ​μm designs, significant impact of CS treatment was observed in 800 ​μm designs where higher vessel properties in CS treated aptamer regions were observed.

### 3.7 Effect of photocrosslinking sequence on guiding spatially defined self-organizing microvasculature

**Fig. 8:**
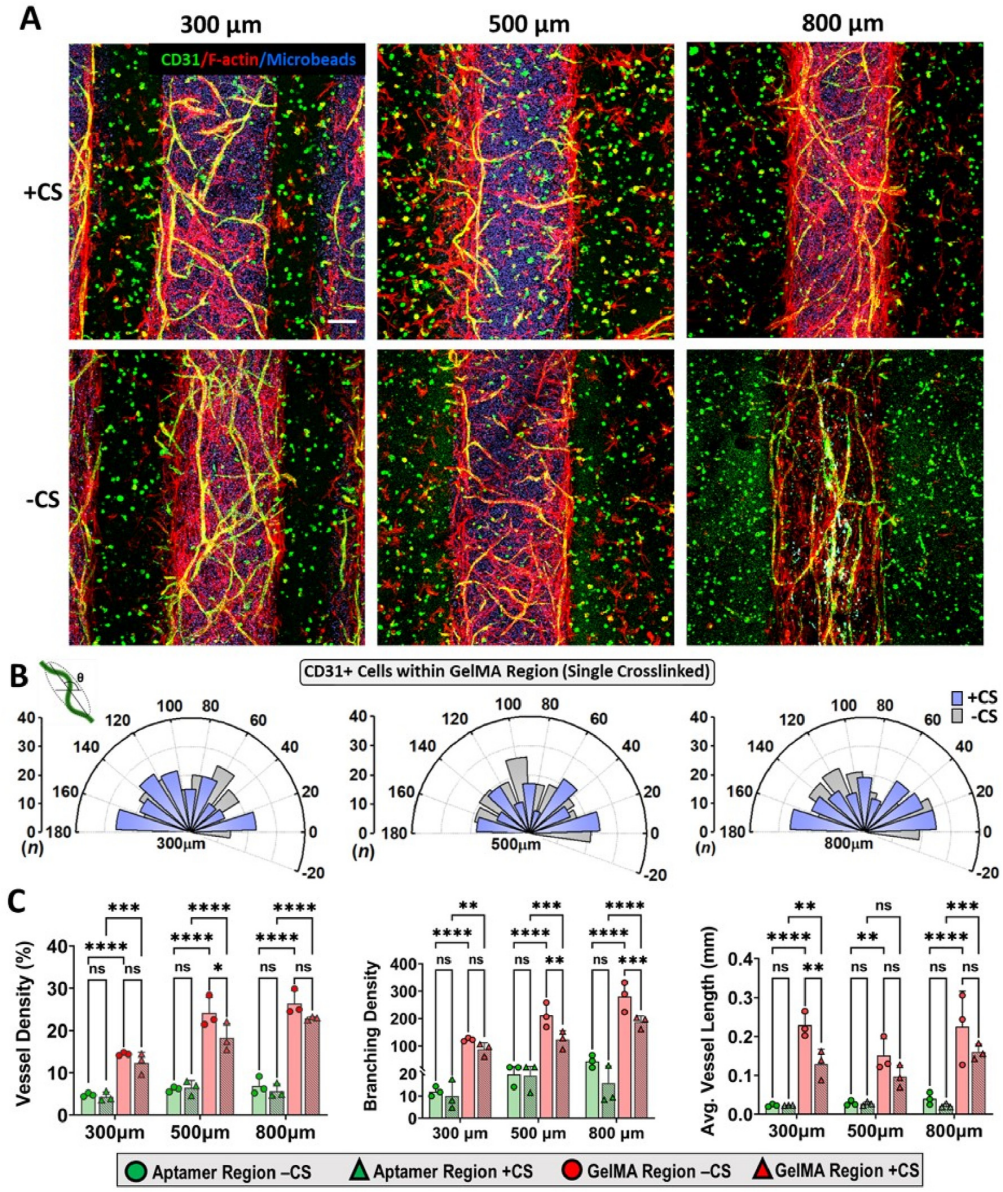
**Effect of photopatterning sequence on controlling self-organizing vascular networks within micropatterns.** (A) Maximum projection confocal Z-stack images showing cell cytoskeletal F-actin (red) and endothelial cells specific CD31 (green) expression within HUVECs/MSCs co-cultured, immunostained bicomponent A_2_G_1_ micropatterns on d10 (with or without CS treatment on d4). The blue color corresponds to fluorescent microbeads present within GelMA region and three micropattern designs were compared with fixed GelMA region (500 ​μm) and varying aptamer region (300/500/800 ​μm), respectively. The scale bar is 100 ​μm. (B) Quantification of CD31 expressing endothelial cells network orientation within GelMA region (single crosslinked) of A_2_G_1_ micropatterns on d10. The polar plots represents “θ” & “n” as endothelial cells network orientation angle and frequency counts binned in 20° increments, respectively. The quantification was performed with three technical replicates, n ​= ​3. (C) CD31 expressing endothelial cells vessel network properties quantification within both regions of A_2_G_1_ micropattern (with or without CS treatment on d4) on d10. The values are represented as mean ​± ​SD, along with individual data points. The calculations were performed with three technical replicates, n ​= ​3. The statistical significance was calculated using two-way ANOVA with Tukey's multiple comparisons test where ∗p ​< ​0.05, ∗∗p ​< ​0.01, ∗∗∗p ​< ​0.001, ∗∗∗∗p ​< ​0.0001 and ns stands for not significant.

Having verified the ability of aptamer-tethered VEGF for spatiotemporally controlling endothelial tubulogenesis within single crosslinked aptamer regions of G_2_A_1_ micropatterns, we next investigated the effect of double exposure during two-step photopatterning in guiding vascular morphogenesis within A_2_G_1_ micropatterns that have double crosslinked patterned aptamer regions. On d10, the patterned GelMA region (single crosslinked) exhibited highly organized and spatially defined CD31^+^ endothelial network formation compared to aptamer regions where minimal cell responses were observed (Fig. 8A). Contrary to G_2_A_1_ micropatterns, where tubular endothelial morphogenesis was observed within the VEGF-rich patterned aptamer region, the A_2_G_1_ micropattern appeared to use aptamer region as the localized VEGF source that facilitated endothelial network formation within neighboring patterned GelMA regions. Upon quantifying the CD31^+^ network orientation within patterned GelMA region, a combinatorial effect of patterned aptamer spacings (i.e., 300/500/800 ​μm) and CS triggered VEGF release was observed (Fig. 8B). Unlike G_2_A_1_ samples, the CS treated A_2_G_1_ micropatterns displayed lowest network frequencies with networks alignment at all angles in 500 ​μm samples [0°-20°(n ​= ​24),40°-60°(n ​= ​22),120°-140°(n ​= ​19)] (Fig. S6A). However, the CS treated 300 ​μm samples showed maximum network frequencies aligned either perpendicular or parallel [160°-180°(n ​= ​26),120°-140°(n ​= ​24),0°-20°(n ​= ​23)], followed by 800 ​μm samples where high network frequencies were aligned perpendicular to the pattern's long axis [160°-180°(n ​= ​26),0°-40°(n ​= ​47)] (Fig. S6A). The non-treated samples showed no obvious changes in network frequency among all designs (Fig. S6B). Moreover, the non-treated 300 ​μm [60°-80°(n ​= ​25),120°-160°(n ​= ​42)] and 500 ​μm samples [0°-20°(n ​= ​21),100°-120°(n ​= ​26),140°-160°(n ​= ​20)] displayed most of the networks aligning parallel to the pattern's long axis, compared to 800 ​μm samples [20°-40°(n ​= ​25),120°-140°(n ​= ​24),160°-180°(n ​= ​23)] (Fig. S6B). Interestingly, high network frequencies aligning parallel to the micropattern's long axis (i.e., 60°-120°) was present in all designs, irrespective of CS treatment (Fig. 8B). In literature, the cells at the periphery of patterns have been shown to elongate and proliferate along the pattern boundary due to the generated traction forces that can dissipate through the soft hydrogel matrices. However, in our results, elongated self-organizing vascular networks were not only confined at the periphery but were well distributed throughout the patterned GelMA regions of 500 ​μm width, highlighting the ability of encapsulated cells present in the middle of the patterns to sense the VEGF availability and participate in the process of tubular endothelial morphogenesis.

The vascular network analysis in micropattern designs exhibited direct relationship between patterned aptamer spacings and properties of the self-organizing vascular network within patterned GelMA region (Fig. 8C). The data revealed that an increase in design spacings resulted in higher vessel properties such as vessel density% and branching density in the following order: 300 ​< ​500<800 ​μm. Furthermore, an overall higher magnitude of vessel properties was observed within non-treated samples compared to CS-treated micropatterns. For instance, in 300 ​μm, the CS-treated samples showed lower vessel density (12.36 ​± ​2.48%) and branching density (88.22 ​± ​23.70) compared to non-treated samples (14.41 ​± ​0.36% & 123.57 ​± ​6.30), respectively. Similarly, 800 ​μm non-treated samples showed highest vessel density (26.44 ​± ​2.97%) and branching density (281.30 ​± ​53.99) than CS-treated ones (22.83 ​± ​0.39% & 187.32 ​± ​22.08), respectively. Likewise, significant differences in 500 ​μm design among CS-treated and non-treated samples in vessel density (18.26 ​± ​3.38% & 24.13 ​± ​3.72%) and branching density (123.37 ​± ​32.96 & 211.67 ​± ​44.67) were observed. However, the 300 ​μm and 800 ​μm designs displayed highest average vessel lengths in non-treated samples (0.229 ​± ​0.03 ​mm & 0.225 ​± ​0.09 ​mm) compared to CS-treated samples (0.129 ​± ​0.03 ​mm & 0.160 ​± ​0.02 ​mm), whereas 500 ​μm designs exhibited average vessel length of 0.151 ​± ​0.04 ​mm in non-treated and 0.097 ​± ​0.02 ​mm in CS-treated samples (Fig. 8C). The observed results show strikingly opposite trends in network properties within G_2_A_1_ and A_2_G_1_ micropatterns. The higher vessel properties in non-treated samples compared to CS treated ones can be attributed to the aptamer-tethered VEGF localization within aptamer regions. Although the exact reason for this anomaly is unclear, it is speculated that the aptamer-tethered VEGF present in aptamer region works as localized source of matrix-bound VEGF that in-turn can establish VEGF concentration gradients stable enough to influence endothelial tubulogenesis in the neighboring patterned GelMA regions. Upon CS triggering, VEGF can be diffused into the surroundings or culture medium, thus reducing the overall VEGF bioavailability within the micropatterns. Similarly, the designs with highest aptamer region spacings (800 ​μm) were observed to elicit maximum network properties, irrespective of CS treatment. Collectively, these results confirm increased aptamer-tethered VEGF localization can facilitate higher vessel properties of spatially defined self-organizing vascular networks within neighboring patterned GelMA regions.

To validate the influence of aptamer-tethered VEGF in eliciting the observed differences in cell behavior within G_2_A_1_ and A_2_G_1_ micropatterns, we investigated G_B2_G_1_ micropatterns as control (without aptamer). Similar to other samples, G_B2_G_1_ micropatterns displayed spatially confined CD31^+^ endothelial tubular morphogenesis within single crosslinked GelMA region compared to double crosslinked GelMA ​+ ​microbead region where minimal cell responses were observed on d10 (Fig. S7A). Interestingly, the single crosslinked patterned GelMA regions of 300 ​μm sample showed polarizing effect of the CD31^+^ network orientation where high network frequencies were aligned parallel to the pattern's long axis [80°-100°(n ​= ​34)] (Fig. S7B). However, the observed polarization tended to decrease with an increase in spacings, which corroborates with literature where patterned endothelial cells within GelMA hydrogels showed maximum alignment in smallest line width (50 ​μm) compared to higher width (300 ​μm) on d5 [35]. The CD31^+^ network analysis revealed overall higher vessel properties such as average vessel length in 300 ​μm (0.108 ​± ​0.024 ​mm) and 500 ​μm (0.105 ​± ​0.023 ​mm) samples than 800 ​μm (0.033 ​± ​0.008 ​mm) samples (Fig. S7C). Similar trends were observed for other vessel properties such as vessel density%, branching density, and total vessel length.

Upon comparing the G_B2_G_1_, A_2_G_1_ and G_2_A_1_ micropatterns, it is evident that opposing trends were observed in G_B2_G_1_ (where smallest spacings resulted increased vessel properties), compared to A_2_G_1_ and G_2_A_1_ micropatterns (where bigger feature spacings, e.g., 500 ​μm and 800 ​μm showed highest vessel properties). This behavior could be attributed to the high local bioavailability of aptamer-tethered VEGF molecules within A_2_G_1_ and G_2_A1 micropatterns where increase in spacings translates as increase in local VEGF concentration, that in turn could enhance endothelial tubular morphogenesis. However, in G_B2_G_1_ micropatterns, due to the absence of aptamer-tethered VEGF molecules, the HUVECs/MSCs are dependent on cell-secreted paracrine factors for establishing the growth factor gradients to guide endothelial network formation. MSCs are able to secrete paracrine factors including VEGF which can significantly enhance endothelial cells migration and proliferation during angiogenesis [39]. Moreover, human endothelial progenitor cells and MSCs co-cultures have been reported to significantly enhance their proliferation and angiogenic potential via the Notch signaling pathway [40]. It is speculated that micropatterns with smallest spacings (300 ​μm) were optimal for establishing the concentration gradients with cell-secreted VEGF that could guide the developing network. However, with an increase in spacings, establishing stable biochemical gradients with cell-secreted VEGF concentration could be difficult leading to reduced network formation. Even though spatially defined vascular networks were achieved within control G_B2_G_1_ micropatterns, the aptamer-tethered VEGF localization have been shown to amplify as well as spatiotemporally control the vessel network properties including alignment of the developing self-organizing vascular networks. Moreover, the aptamer-tethered VEGF localization showed accelerated effect on vascular morphogenesis, where stable tubular networks were formed by d10, as opposed to previously reported studies where stabilized micro-capillaries with lumens were formed within HUVECs/MSCs co-cultured gelatin-modified hydrogels by d14 of culture [41]. Similarly, Barrs et al., demonstrated luminal microvascular network formation of approximate 9 ​μm diameter, within HUVECs/human adipose-derived stem cells co-cultured, alginate hydrogels functionalized with cell-adhesive (RGD) and VEGF-mimicking (MMPQK) peptides after 14 ​d of culture [42].

It is worth noting that the double photo-exposed regions within all micropatterns (A_2_G_1_, A_2_G_1_ & G_B2_G_1_), irrespective of CS treatment, displayed minimal cell responses such as spreading, sprouting and directionality throughout the culture duration. Even though cell viability was high in all regions, it is speculated that double photo-exposure during the two-step photopatterning process could lead to increased free radical formation that in-turn can have detrimental effect on cells. Similar behavior has been previously observed with UV based photoinitiators. Lin et al., reported that by increasing UV exposure time from 15s to 45s, significant decrease in cell spreading was observed within human endothelial colony forming cells and MSCs encapsulated GelMA hydrogels *in vivo* [43]. High UV exposure time regressed the vascular morphogenesis *in vivo* where lower number of perfused blood vessels (145 lumens/mm^2^) were observed within 30s UV exposed samples compared to 15s samples (374 lumens/mm^2^), whereas no obvious blood vessels were observed within GelMA hydrogels UV exposed for more than 45s [43]. Though high cell viability and metabolic activities have been reported with Ru/SPS (0.2/2 ​mM/mM)+visible light system for up to 15min within human articular chondrocytes encapsulated GelMA hydrogels [44], contrasting results were observed within the present study.

Taken together, these experiments unravel aptamer-tethered VEGF localization as a versatile tool for spatiotemporally guiding the self-organizing vascular network properties including alignment within 3D engineered matrices. Importantly, prolonged light exposure during photopatterning inhibited the cellular responses such as spreading, sprouting, migration and proliferation within cell-laden bicomponent patterns. Future work should therefore prudently consider advanced biofabrication techniques such as 3D bioprinting to overcome the limitation of double exposure for patterning spatially defined localized aptamer structures. Given the programmable nature of VEGF presentation and its effect on vascular morphogenesis, this study suggests that aptamer-tethered VEGF platform can be utilized for regulating network remodeling *in-situ*. Consequently, the platform could further be exploited for dynamic presentation of multiple growth factors, for example, VEGF and PDGF-BB, to mimic complex processes such as angiogenesis *in vitro* for vascularized engineered tissues.

## 4 Conclusion

Our study highlight the potential of aptamer-tethered VEGF within biofabricated GelMA based bicomponent micropatterns using visible-light photoinitiators (Ru/SPS) as a dynamic and versatile tool for VEGF localization that can spatiotemporally modulate vascular morphogenesis *in vitro*. Its ability to retain high aptamer and display high molecular recognition among aptamer-CS_F_ as well as exhibit maximum VEGF sequestration from the culture medium within patterned aptamer regions, provide unpreceded leverage to this approach. Despite the stiffness gradient within micropatterns, high bioactivity within HUVECs/MSCs encapsulated micropatterns were observed till d5, irrespective of CS treatment. Moreover, cell-laden micropatterns displayed unique control over cell orientation till d5, in the direction of VEGF gradient established at the interface among both regions. With dramatically enhanced spatial control over vascular network formation compared to present growth factor delivery approaches, the aptamer-tethered VEGF platforms can further control vascular network organization over time using CS as an external trigger within G_2_A_1_ and A_2_G_1_ micropatterns. Furthermore, the comparison of micropatterns with different spatial spacings (300/500/800 ​μm) in G_2_A_1_ and A_2_G_1_ samples, revealed 500 ​μm and 800 ​μm spacings as optimal for engineering vessel networks with enhanced vessel properties. More importantly, the observed unique cell behavior and vascular network organization was found to be specifically linked to the aptamer-tethered VEGF localization and was not replicable within pristine GelMA micropatterns (G_B2_G_1_). These promising characteristics of aptamer-tethered VEGF based platforms opens up new avenues for *in-situ* manipulation of vascular networks dynamically after biofabrication as well during remodeling within engineered tissues. Taken together, this study demonstrates aptamer-tethered VEGF patterning as a versatile tool for dynamic growth factor presentation within engineered matrices that can spatiotemporally manipulate self-organizing microvasculature *in-situ*.

## Credit author statement

Deepti Rana: Conceptualization, Methodology, Investigation, Formal analysis, Writing – original draft, Writing – review & editing. Prasanna Padmanaban: Formal analysis, Investigation. Malin Becker: Formal analysis, Investigation. Fabian Stein: Formal analysis, Investigation. Jeroen Leijten: Resources, Formal analysis. Bart Koopman: Supervision, Project administration. Jeroen Rouwkema: Conceptualization, Writing – review & editing, Supervision, Project administration, Funding acquisition.

## Declaration of competing interest

The authors declare that they have no known competing financial interests or personal relationships that could have appeared to influence the work reported in this paper.

## Data Availability

Data will be made available on request.
