# Supplementary material for: Spatial control of self-organizing vascular networks with programmable aptamer-tethered growth factor photopatterning
Source: Mater Today Bio. 2023 Jan 20;19:100551. doi: 10.1016/j.mtbio.2023.100551 (PMC9898740; doi:10.1016/j.mtbio.2023.100551)
Supplement: Multimedia component 1 [file mmc1.docx]

Spatial control of self-organizing vascular networks with programmable aptamer-tethered growth factor photopatterning

Deepti Rana^1^, Prasanna Padmanaban^1^, Malin Becker^2^, Fabian Stein^1^, Jeroen Leijten^2^, Bart Koopman^1^ and Jeroen Rouwkema^1^*

*^1^Department of Biomechanical Engineering, Technical Medical Centre,*

*Faculty of Engineering Technology, University of Twente, 7522NB Enschede, The Netherlands
^2^Department of Developmental BioEngineering, Faculty of Science and Technology,*

*Technical Medical Centre, University of Twente, 7522NB Enschede, The Netherlands*

**Corresponding Author E-mail:* [*j.rouwkema@utwente.nl*](mailto:j.rouwkema@utwente.nl)

Supporting Information

1. Supplementary Methods Section

S1. Materials

Type A 300 bloom porcine skin gelatin (G1890-500G, Sigma Aldrich), Methacrylic anhydride (MA, 276685-500ML, Sigma Aldrich), Dulbecco’s phosphate buffered saline (DPBS, D8537-500ML, Sigma Aldrich), Fisherbrand™ regenerated cellulose dialysis tubing (12-14 kDa, 21-152-14, Fisher Scientific), bovine serum albumin (BSA, A9418, Sigma Aldrich), tris(2,2‘-bipyridyl)dichloro-ruthenium (II) hexahydrate (224758, Sigma), sodium persulfate (S6172, Sigma), VEGF specific 5’acrydite-modified aptamer (47-nt, DNA, IDT), complementary sequence (CS, 46-nt, DNA, IDT), 5’Alexa Fluor 488 modified complementary sequence (CS_F_, 46-nt, DNA, IDT), nuclease free water (11-04-02-01, IDT), 3-(trimethoxysilyl)propyl methacrylate (TMSPMA, 440159, Sigma-Aldrich), photomask films (custom-made, Selba S.A.), Fluoro-Max dyed blue aqueous fluorescent particles (2µm diameter, B0200, Thermo Scientific), Alexa Fluor® 647 Anti-VEGFA antibody [EP1176Y] (ab206887, abcam), human umbilical vein endothelial cells (HUVECs, C2519A, Lonza), human mesenchymal stromal cells (MSC, PT-2501, Lonza), α –MEM medium (+nucleosides, 22571-020, Gibco), fetal bovine serum (FBS, F7524, Sigma), GlutaMax™ supplement (35050061, Gibco), penicillin-streptomycin (pen/strep, 15140-122, Gibco), L-abscorbic acid (A8960, Sigma Aldrich), trypsin-EDTA 0.25% (+phenol red, 25200072, Gibco), vascular endothelial growth factor 165 human (VEGF, H9166, Sigma Aldrich), Gibco™ FGF-Basic AA 1-155 recombinant human protein (bFGF, PHG0264, Fisher Scientific), endothelial cell basal medium 2 (EGM 2, C-22211, PromoCell), endothelial cell growth medium 2 supplement pack (EGM 2, C-39211, PromoCell), PDMS silicone elastomer (2401673921, Sylgard), live/dead cell double staining kit (04511, Sigma Aldrich), formaldehyde solution (F8775-25ML, Sigma Aldrich), Triton™ X-100 (T8787, Sigma Aldrich), Invitrogen™ Alexa Fluor™ 647 Phalloidin (A22287, Fisher Scientific), Invitrogen™ hoechst 33342 (H1399, Fisher Scientific), CD31 monoclonal antibody (HEC7, mouse, MA3100, Invitrogen), IgG (H+L) cross-adsorbed goat anti-mouse secondary antibody, Alexa Fluor® 488 (A11001, Invitrogen), Corning® Costar® ultra-low attachment well plates were used for all cell culture experiments (CLS3471 Sigma).

S2. Modelling of the free VEGF release using COMSOL Multiphysics software

The model for the analysis and prediction of the free VEGF diffusion was developed under the following assumptions:

1. The system is in equilibrium at the beginning of the simulation (t = 0 h)
2. Free VEGF transports only results from the diffusion
3. Freely diffusing VEGF molecules are the only mobile entities in the system
4. Aptamers are bound onto the hydrogels and tethers free VEGF molecules
5. Upon CS addition, the free VEGF molecules released in the system
6. Higher amount of free VEGF present in the aptamer functionalized hydrogel compared to GelMA hydrogel compartment
7. Free VEGF diffuses from aptamer functionalized region to bulk GelMA regions.
8. Similar to actual sample dimensions, the aptamer and GelMA regions were simulated as rectangles of 1 mm length and 400 µm breadth having placed as aptamer line in middle and GelMA lines on both sides with an interface, in 2D simulations.
9. For 3D simulations, three lines as continues regions placed next to each other in cuboid shape with each region having the following dimensions were used: x=500 µm, y=8 mm & z=800 µm.

The spatiotemporal change in the free VEGF concentration within the hydrogel due to the diffusion over time is given by following equations:

$\frac{\boldsymbol{\partial}\boldsymbol{C}_{\boldsymbol{VEGF-aptamer}}}{\boldsymbol{\partial t}}\boldsymbol{=}\boldsymbol{D}_{\boldsymbol{VEGF+CS}}\boldsymbol{\nabla}^{\boldsymbol{2}}\boldsymbol{C}_{\boldsymbol{VEGF-aptamer}}$ [with CS treatment] – (eq. 1)

$$\frac{\boldsymbol{\partial}\boldsymbol{C}_{\boldsymbol{VEGF-GelMA}}}{\boldsymbol{\partial t}}\boldsymbol{=}\boldsymbol{D}_{\boldsymbol{VEGF-CS}}\boldsymbol{\nabla}^{\boldsymbol{2}}\boldsymbol{C}_{\boldsymbol{VEGF-GelMA}}$$

 [without CS treatment] – (eq. 2)

Where parameters such as *C_VEGF-aptamer_* and *C_VEGF-GelMA_* denotes free VEGF concentration in aptamer and GelMA regions at *t* = 0 hr, respectively. Additionally, *D_VEGF+CS_* and *D_VEGF-CS_* stands for free VEGF diffusivity in the presence and absence of CS throughout the micropattern, respectively **(Table S2)**. In both 2D and 3D simulations, we used transport of diluted species physics module in COMSOL and physics controlled extremely fine mesh conditions was applied to the models for computing free VEGF diffusion.

1. Supplementary Figures


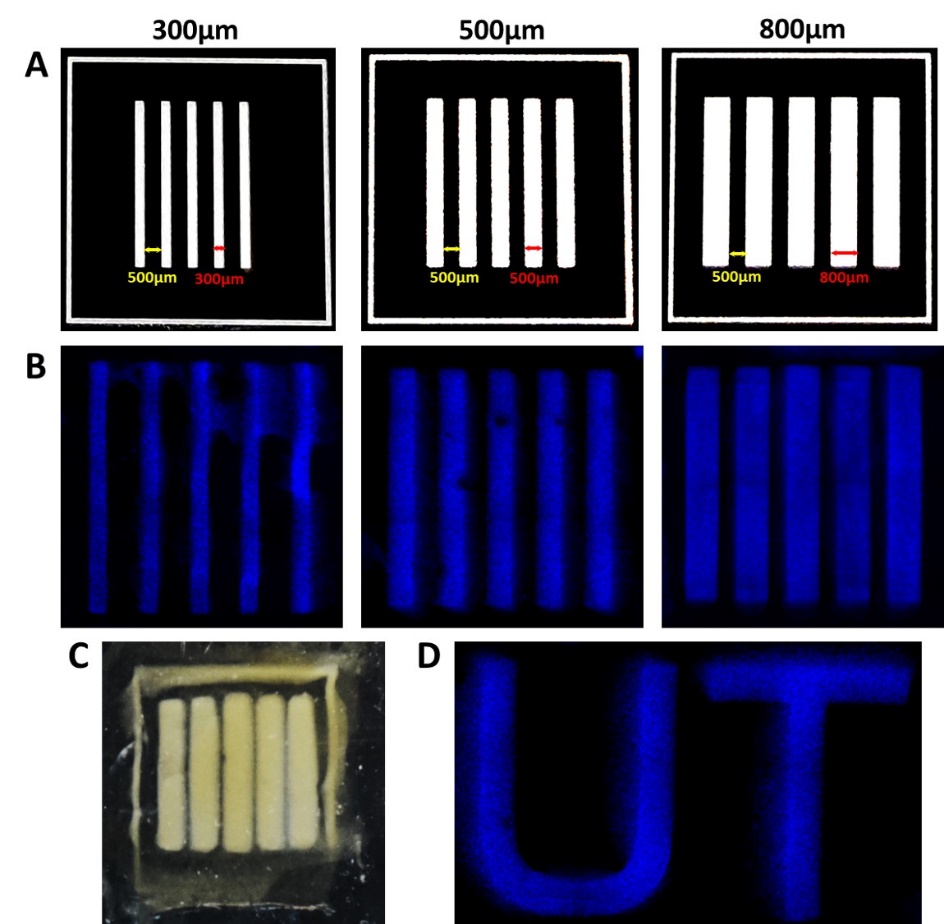


Figure S1. Photopatterning designs. (A) The photomask films of the different micropatterns used in the present study. The photomask design highlights constant spacing width (yellow, 500 µm) and varying beam width (red) i.e., 300 µm, 500 µm & 800 µm. (B) The fluorescence microscopic stitched image of the corresponding micropattern fabricated via two-step photocrosslinking method. The blue fluorescence microbeads were mixed with GelMA pre-polymer for the 1^st^ crosslinking cycle followed by plain GelMA for 2^nd^ crosslinking cycle. (C) Macroscopic photograph of the micropattern (800 µm) showing microbeads mixed GelMA patterns overlaid with transparent plain GelMA. (D) The fluorescence microscopic stitched image of the UT logo micropattern fabricated same as above.


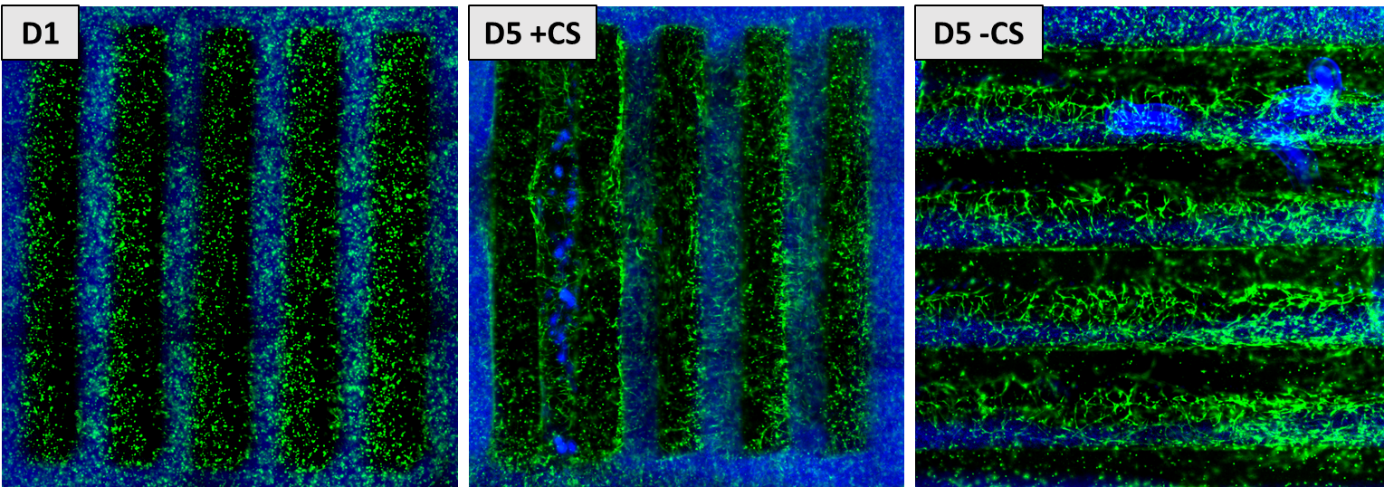


**Figure S2. Cell viability of micropattern with/without CS treatment on d 1 and d 5.** Live/Dead stained fluorescent microscopic stitched images (at 10x magnification) of HUVECs/MSCs co-cultured A_2_G_1_ micropattern (aptamer line-500 µm; GelMA line, blue-500 µm) with VEGF specific aptamer-functionalized hydrogel on d 1. To trigger the VEGF release from the patterned aptamer region, the micropatterns were incubated with CS on d 4. (B) The stitched images of micropatterns treated with CS and (C) without CS on d 5. The green and red color represents live and dead cells, respectively. Blue color corresponds to the GelMA region mixed with blue fluorescent microbeads in the micropattern.


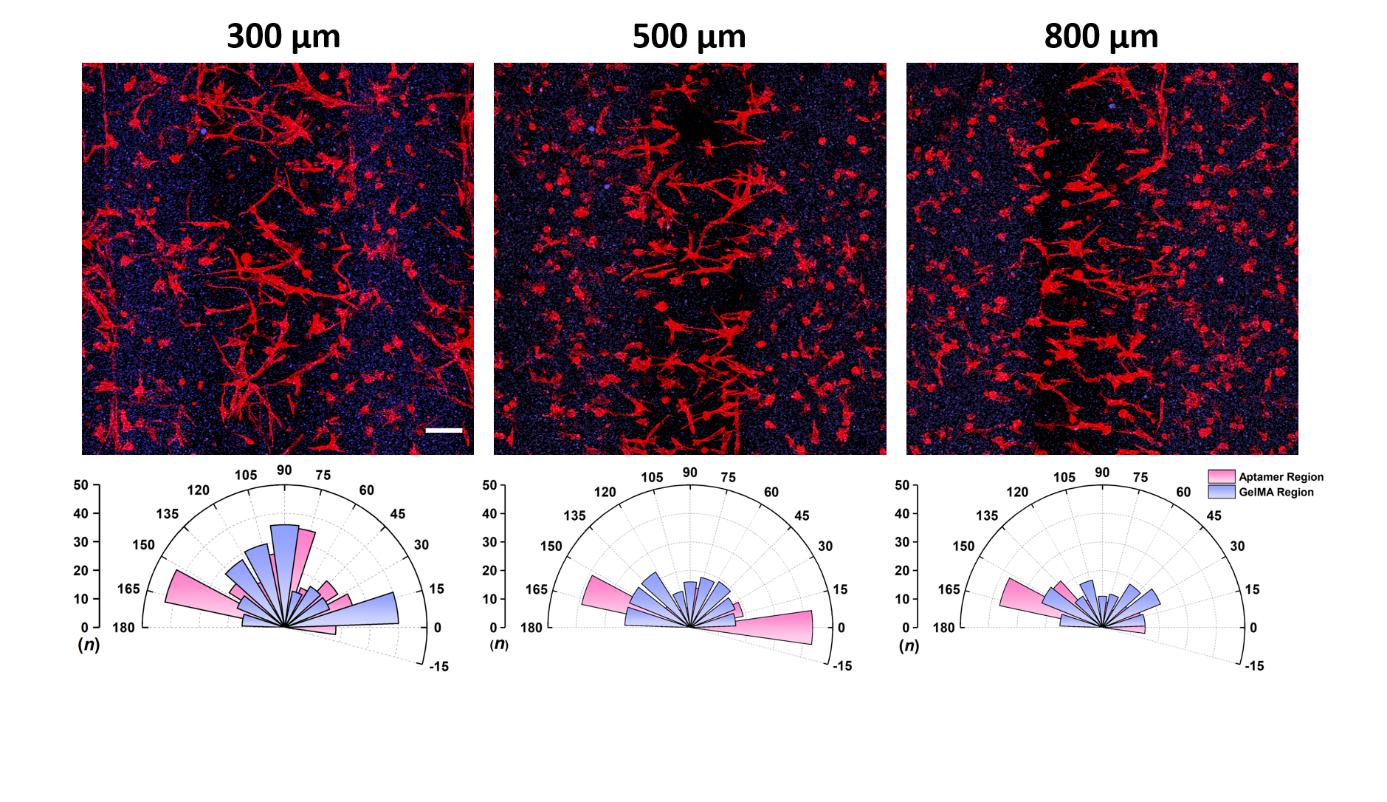
Figure S3. Spatially confined cellular orientation within micropatterns on d 3. Maximum projection of confocal Z-stack images showing cell cytoskeletal F-actin (red) stained HUVECs/MSCs co-cultured bicomponent G_2_A_1_ micropatterns on d 3. The samples were quantified for individual cell orientation within aptamer and GelMA regions of the micropattern using F-actin stained microscopic images. The data is represented as polar plot where “θ” & “r” represents cell orientation angle and frequency counts binned in 15º increments, respectively. The quantification was performed with three technical replicates, n=3. The blue color corresponds to fluorescent microbeads present in GelMA region. For comparison, three micropattern designs were studied that have fixed aptamer region (500 µm) but varies in GelMA region (300 µm, 500 µm & 800 µm), respectively. The scale bar is 100 µm.


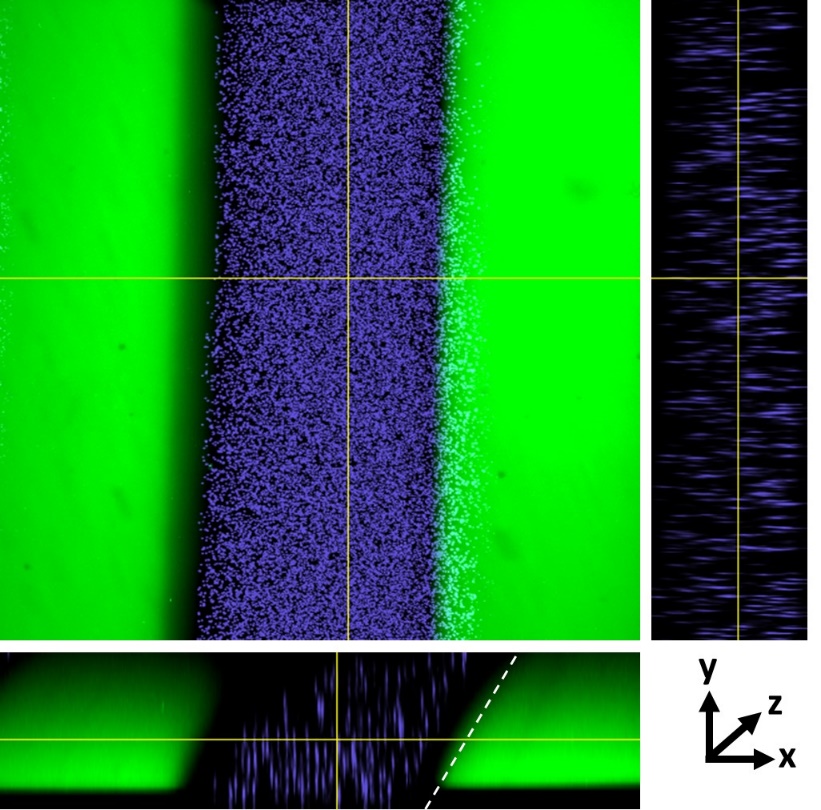


Figure S4. Orthogonal view of bicomponent micropattern. The confocal Z-stack (z=310 µm) in orthogonal view highlighting the photopatterned regions overlapping at an tilted angle throughout the micropattern depth, as marked with white dotted line. The observed effect is caused due to the deflection of light passing through the photomask during photopatterning process.


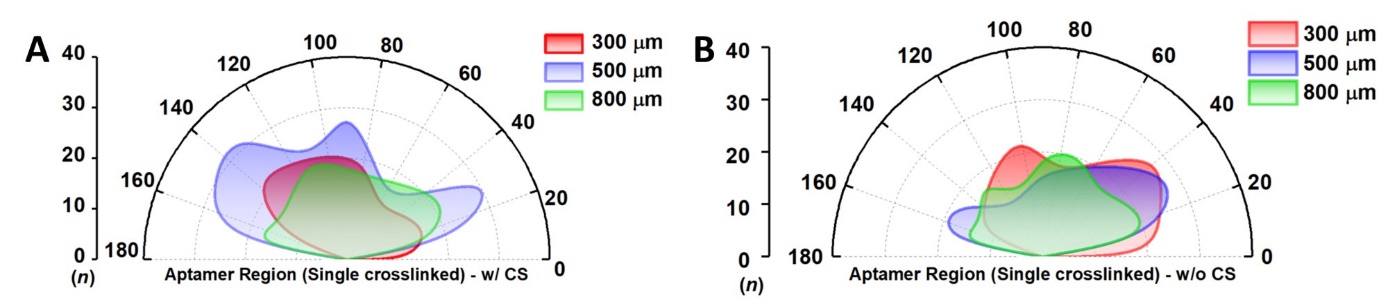


Figure S5. CD31 expressing endothelial cells based microvascular network orientation within aptamer region of the G_2_A_1_ micropatterns (300 µm, 500 µm & 800 µm) on d 10. The samples treated with and without CS on d 4 were compared. The CD31+ network orientation data as displayed in Fig. 7B, rearranged to highlight the effect of CS treatment on network orientation among different GelMA spacings. The polar plots represents “θ” & “r” as endothelial cells network orientation angle and frequency counts binned in 20º increments, respectively. The quantification was performed with three technical replicates, n=3.


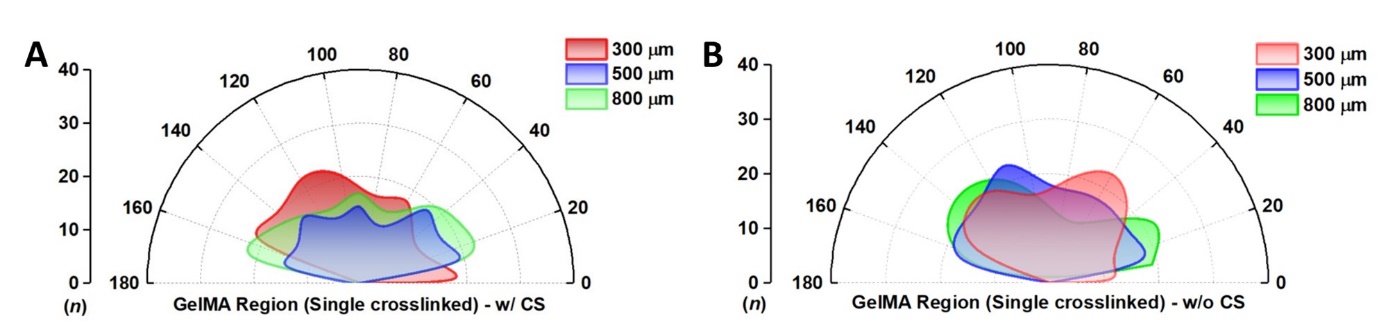


Figure S6. CD31 expressing endothelial cells based microvascular network orientation within GelMA region of the A_2_G_1_ micropatterns (300 µm, 500 µm & 800 µm) on d 10. The samples treated with and without CS on d 4 were compared. The CD31+ network orientation data as displayed in Fig. 8B, rearranged to highlight the effect of CS treatment on network orientation among different aptamer spacings. The polar plots represents “θ” & “r” as endothelial cells network orientation angle and frequency counts binned in 20º increments, respectively. The quantification was performed with three technical replicates, n=3.


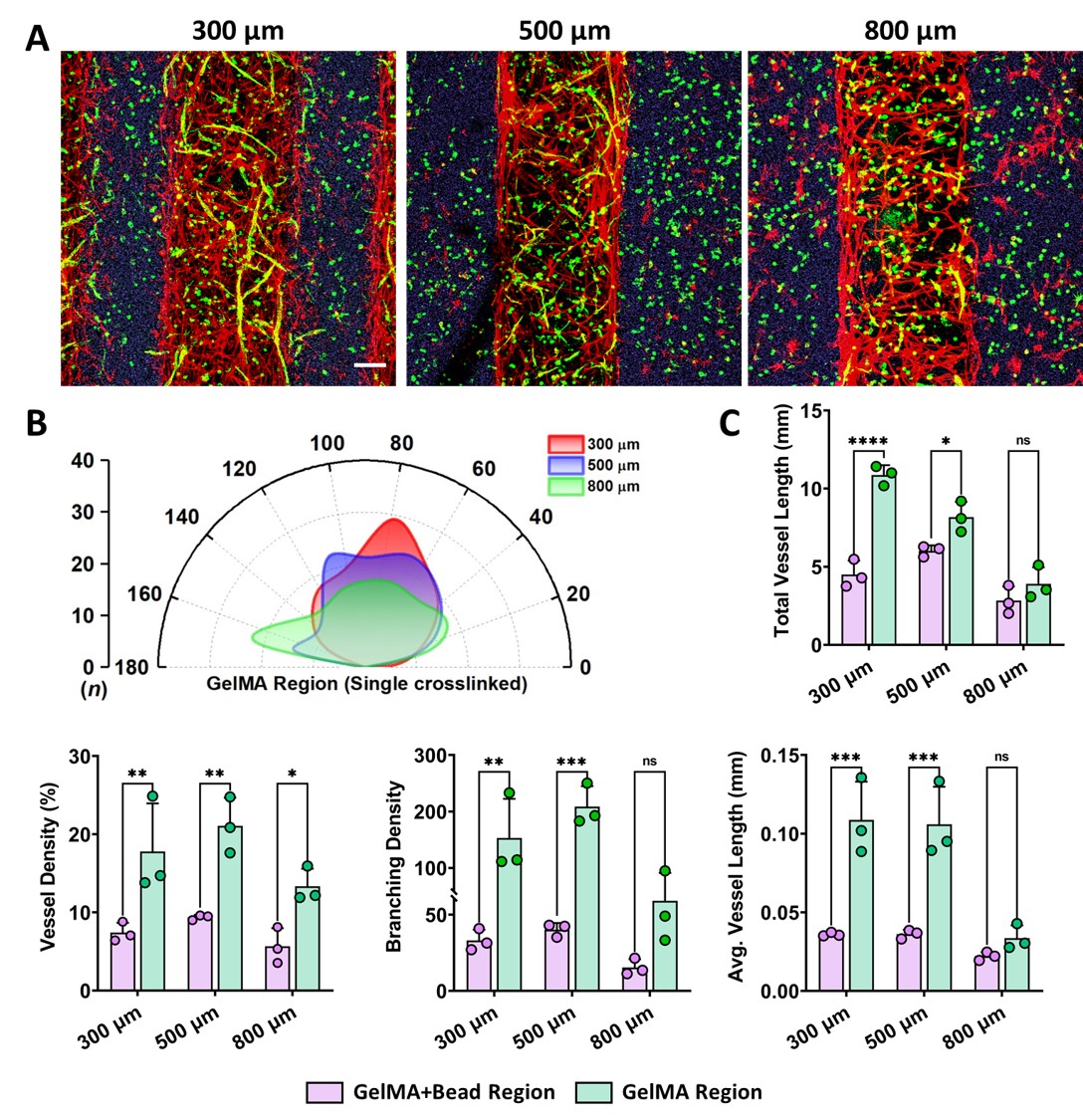


**Figure S7.** **Patterned microvascular network formation within cell-laden only GelMA based micropatterns on d 10.** (A) Maximum projection confocal Z-stack images showing cell cytoskeletal F-actin (red) and endothelial cells specific CD31 (green) expression within HUVECs/MSCs co-cultured, immunostained bicomponent GelMA based micropatterns (G_B2_G_1_) on d 10 as control. The blue color corresponds to fluorescent microbeads present within double photocrosslinked GelMA region. For comparison, three micropattern designs were studied that have fixed GelMA region (500 µm) and varies GelMA + blue microbeads region (300 µm, 500 µm & 800 µm), respectively. The scale bar is 100 µm. (B) Quantification of CD31 expressing endothelial cells network orientation within single crosslinked GelMA region of G_B2_G_1_ micropatterns (300 µm, 500 µm & 800 µm). The polar plots represents “θ” & “r” as endothelial cells network orientation angle and frequency counts binned in 20º increments, respectively. The quantification was performed with three technical replicates, n=3. (C) CD31 expressing endothelial cells vessel network properties quantification within both regions of G_B2_G_1_ micropattern using Angiotool software (NIH) on d 10. The values are represented as mean ± SD, along with individual data points. The calculations were performed with three technical replicates, n=3. The statistical significance was calculated using two-way ANOVA with Tukey’s multiple comparisons test where *p<0.05, **p<0.01, ***p<0.001, ****p<0.0001 and ns stands for not significant.

**Supporting Tables & Video**

**Table S1.** Aptamer sequences and their characteristics used in the present study. Tm denotes the melting temperature (50 mM NaCl), MW is molecular weight and N signifies the number of nucleotides.

| Aptamer | Sequence (5’🡪 3’) | Tm | MW | N |
| --- | --- | --- | --- | --- |
| VEGF Specific Aptamer | /5Acryd/CGA TCG TAT CAG TCC ACA AGC CCG TCT TCC AGA CAA GAG TGC AGG GC | 70.8 °C | 14665.6 | 47 |
| Complementary Sequence (CS) | CGC CCT GCA CTC TTG TCT GGA AGA CGG GCT TGT GGA CTG ATA CGA TCG | 71.3 °C | 14791.6 | 48 |
| Fluorescently labelled CS (CS_F_) | /5Alexa488N/CGC CCT GCA CTC TTG TCT GGA AGA CGG GCT TGT GGA CTG ATA CGA TCG | 71.3 °C | 15487.2 | 48 |

**Table S2.** Parameters used for the reaction-diffusion model of free VEGF release within aptamer functionalized hydrogels.

| **Parameters** | **Values** |
| --- | --- |
| VEGF concentration in Aptamer region at *t* = 0hr **[**$\boldsymbol{C}_{\boldsymbol{VEGF-aptamer}}\boldsymbol{]}$ | 0.25 nM |
| VEGF concentration in GelMA region at *t* = 0hr $\boldsymbol{[C}_{\boldsymbol{VEGF-GelMA}}\boldsymbol{]}$ | 0.012 nM |
| VEGF Diffusivity in the absence of CS $\boldsymbol{[D}_{\boldsymbol{VEGF-CS}}\boldsymbol{]}$ | 0 m^2^/s |
| VEGF Diffusivity in the presence of CS $\boldsymbol{[D}_{\boldsymbol{VEGF+CS}}\boldsymbol{]}$ | 1.8 x 10^-13^ m^2^/s[1] |

**Video S1.** 3D projection of confocal Z-stacks showing bicomponent A_2_G_1_ micropattern having both aptamer and GelMA regions (aptamer line, green-500 µm; GelMA line, blue-500 µm) after 24 hr incubation with CS_F_ at 37ºC. To highlight the interface between acrydite-aptamer and GelMA regions, blue fluorescent particles (2 µm diameter) were mixed with GelMA pre-polymer solution. The 3D image confirms the homogenous sequestration of CS_F_ throughout the micropattern thickness (z = 310µm).

**Video S2.** *In-silico* analysis of free VEGF diffusion from aptamer region to GelMA region in the absence of CS during 144 hr (d6) of experiment.

**Video S3.** *In-silico* analysis of free VEGF diffusion from aptamer region to GelMA region in the presence of CS during 144 hr (d6) of experiment.

**Video S4.** 3D projection of confocal Z-stack showing F-actin and CD31 immunostained HUVECs/MSCs at the interface between aptamer and GelMA (blue microbeads) regions at 60x magnification. The 3D projection shows lumen-like vessel structure of the newly developing vascular network (stack thickness, z = 42 µm).

**References**

[1] Abune L, Zhao N, Lai J, Peterson B, Szczesny S and Wang Y 2019 Macroporous hydrogels for stable sequestration and sustained release of vascular endothelial growth factor and basic fibroblast growth factor using nucleic acid aptamers *ACS Biomaterials Science and Engineering*
